# Her9 controls the stemness properties of hindbrain boundary cells

**DOI:** 10.1242/dev.203164

**Published:** 2025-01-02

**Authors:** Carolyn Engel-Pizcueta, Covadonga F. Hevia, Adrià Voltes, Jean Livet, Cristina Pujades

**Affiliations:** ^1^ Department of Medicine and Life Sciences, Universitat Pompeu Fabra, 08003 Barcelona, Spain; ^2^ Sorbonne Université, INSERM, CNRS, Institut de la Vision, 75012 Paris, France

**Keywords:** Hindbrain boundaries, Neural progenitors, Zebrafish, Multicolor clonal analysis, *her9*, zebrabow2.0

## Abstract

The different spatiotemporal distribution of progenitor and neurogenic capacities permits that brain regions engage asynchronously in neurogenesis. In the hindbrain, rhombomere progenitor cells contribute to neurons during the first neurogenic phase, whereas boundary cells participate later. To analyze what maintains boundary cells as non-neurogenic progenitors, we addressed the role of Her9, a zebrafish Hes1-related protein. *her9* expression is temporarily sustained in boundary cells independently of Notch at early embryonic stages, while they are non-neurogenic progenitors. Complementary functional approaches show that Her9 inhibits the onset of Notch signaling and the neurogenic program, keeping boundary cells as progenitors. Multicolor clonal analysis combined with genetic perturbations reveal that Her9 expands boundary progenitors by promoting symmetric proliferative and preventing neurogenic cell divisions. Her9 also regulates the proliferation of boundary cells by inhibiting the cell cycle arrest gene *cdkn1ca* and interplaying with Cyclin D1*.* Moreover, *her9* is enriched in hindbrain radial glial cells at late embryonic stages independently of Notch. Together these data demonstrate that Her9 maintains the stemness properties of hindbrain boundary progenitors and late radial glial cells, ensuring the different temporal distribution of neurogenic capacities within the hindbrain.

## Teaser

**Summary:** Zebrafish Her9 keeps boundary cells in the hindbrain as non-neurogenic progenitors by inhibiting Notch signaling and promotes their proliferation by controlling cell cycle regulators*.*

## INTRODUCTION

During early brain development, neuroepithelial cells, a primary form of neural stem cells, proliferate by symmetric cell divisions to contribute to the growth of the neural tube. Subsequently, neuroepithelial cells transition into radial glial cells that divide asymmetrically, giving rise to neurons while maintaining the stem cell pool. Thus, timely shifts of cell division modes are crucial to form a functional brain. In the hindbrain, the embryonic brainstem, the orchestrated spatiotemporal distribution of progenitor and neurogenic capacities results in hindbrain territories engaging asynchronously in neurogenesis (Belmonte-Mateos et al., 2023; Gonzalez-Quevedo et al., 2010; Hevia et al., 2022; Nikolaou et al., 2009; Peretz et al., 2016; Voltes et al., 2019). This asynchrony relies on a spatial organization that results from hindbrain segmentation and gives rise to seven transient rhombomeres (Kiecker and Lumsden, 2005). At the interface between rhombomeres, there is the specification of the boundary cell population (Guthrie and Lumsden, 1991; Lumsden and Keynes, 1989), which displays a specific gene expression (Cheng et al., 2004; Letelier et al., 2018) and distinct functions during development (Pujades, 2020). Rhombomeres engage early in neurogenesis (Amoyel et al., 2005; Nikolaou et al., 2009), whereas boundary cells participate later (Hevia et al., 2022; Peretz et al., 2016; Voltes et al., 2019). This distinct neurogenic commitment is mainly regulated by Notch signaling. During the early neurogenic phase, the Notch pathway is highly active in the rhombomere compartments (Nikolaou et al., 2009), while inactive in the hindbrain boundary regions, which are composed of neuroepithelial cells dividing in a proliferative symmetric mode (Hevia et al., 2022; Voltes et al., 2019). Later, Notch3 activity triggers the switch of boundary cells to radial glia progenitors that divide asymmetrically (Hevia et al., 2022). Yap/Taz activity maintains boundary cells as highly proliferative progenitors at early embryonic stages (Voltes et al., 2019). However, the mechanisms preventing boundary cells from transitioning to neurogenesis and the cell cycle regulators controlling their proliferative capacity are still unknown.

The Hes family of basic helix-loop-helix (bHLH) transcriptional repressors is key for the balance between cell differentiation and proliferation (Kageyama et al., 2008). Although Hes factors are the main effectors of Notch signaling in neurogenesis (Ohtsuka et al., 1999), there is a set of Hes genes for which expression is independent of Notch (Geling et al., 2003; Ninkovic et al., 2004). In teleosts, Hes factors [Her5 and Him (also known as Her11)] maintain neural progenitors at the mid-hindbrain boundary by inhibiting neurogenesis independently of Notch (Geling et al., 2003; Ninkovic et al., 2004). In mammals, brain boundaries express high Hes1 in contrast with low Hes1 compartments (Baek et al., 2006). Loss of only *Hes1*, or in combination with *Hes3* and *Hes5*, results in ectopic proneural gene expression and premature neuronal differentiation in the embryonic brain (Cau et al., 2000; Hatakeyama et al., 2004; Hirata et al., 2001; Ishibashi et al., 1995; Ohtsuka et al., 1999), whereas high *Hes1* expression represses neurogenesis and decreases proliferation (Baek et al., 2006; Ohtsuka and Kageyama, 2021; Sueda et al., 2019). Accordingly, misexpression of Hes1 in telencephalic neural progenitors leads to inhibition of *Neurog2*, *Delta1*, and cell cycle-related genes (Shimojo et al., 2008).

To further analyze the mechanisms that maintain hindbrain boundary cells as proliferative progenitors and restrict them from undergoing neurogenesis, we addressed the role of a zebrafish Hes1-related protein, Her9. We demonstrate that *her9* is temporally enriched independently of Notch in boundary cells when they are non-neurogenic progenitors. Functional manipulation of Her9 using complementary strategies reveals that it represses the onset of Notch signaling and the neurogenic program, thus keeping boundary cells in the progenitor state. Functional multicolor clonal analyses show that Her9 controls the expansion of the boundary progenitor population by promoting symmetric proliferative while blocking neurogenic cell divisions. Furthermore, Her9 inhibits the cell cycle arrest gene *cdkn1ca* and cooperates with Cyclin D1 to control the proliferative capacity of boundary cells. *her9* is also highly expressed in distinct late neural progenitor populations independently of Notch when neurogenesis has ceased in the hindbrain. Thus, we propose Her9 as a key player for maintaining the progenitor status through space and time during hindbrain development.

## RESULTS

### *her9* is temporarily enriched in the hindbrain boundaries independently of Notch signaling

**Fig. 1.**
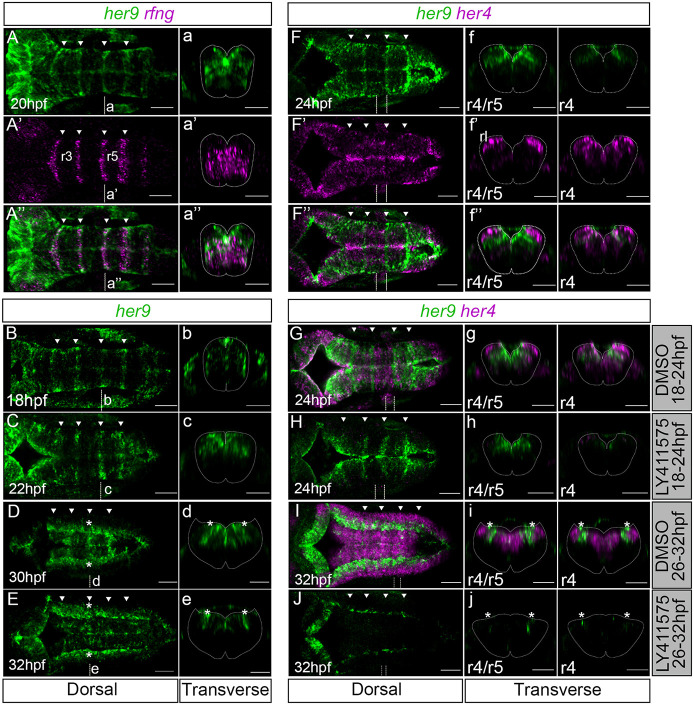
***her9* is temporarily enriched in the hindbrain boundaries independently of Notch at early embryonic stages.** (A-J) *her9 in situ* hybridization with *rfng* (A′,A″) and *her4* (F′,F″,G-J) at the indicated developmental stages. (G-J) Embryos treated with DMSO (G,I) or LY411575 (H,J) (18-24 hpf: *n*=4/4 DMSO versus *n*=14/14 LY411575; 26-32 hpf: DMSO *n*=10/10 versus LY411575 *n*=8/9). (A-A″,B-E,F-F″,C-J) Dorsal maximal intensity projections of the hindbrain with anterior to the left. (a-a″,b-e,f-f″,g-j) Transverse views of r4/r5 boundary or r4. Arrowheads indicate the position of the hindbrain boundaries. Dotted lines delimitate the contour of the neural tube. Dashed vertical lines indicate the levels of the corresponding transverse projections in other panels. hpf, hours post fertilization; r, rhombomere; rl, rhombic lip. Scale bars: 50 µm.

To explore the role of Her9 on zebrafish hindbrain boundary cells, we first performed *in situ* hybridization to detect expression of *her9* and well-described hindbrain boundary markers such as *rfng* and *foxb1a* (Cheng et al., 2004). At early embryonic stages, *her9* expression was enriched in hindbrain boundaries where it largely overlapped with boundary markers, whereas lower levels were observed within the rhombomeres (Fig. 1A-A″,a-a″; Fig. S1A-A″,B-B″,a-a″,b-b″). The spatiotemporal analysis revealed that *her9* in the boundaries was expressed at 18 hours post-fertilization (hpf) (Fig. 1B,b) coinciding with the onset of boundary-specific gene expression (Letelier et al., 2018). This sustained *her9* expression in the boundaries' progenitor domain was maintained upon ventricle formation (22-30 hpf; Fig. 1C,D,c,d). From 26 hpf onwards, *her9* became also enriched in two lateral stripes along the anteroposterior (AP) axis (Fig. 1D,E,d,e; Fig. S1A,B,B′,A″-B″; a,b,b′,a″-b″). The sustained *her9* expression in the boundaries was lost from 32 hpf onwards, and *her9* became homogeneously expressed in boundaries and rhombomere compartments (Fig. 1E,e; Fig. S1D,d). Next, we compared *her9* expression to *her4*, which is a classical Notch effector expressed in neurogenic progenitors (Takke et al., 1999), before and after the onset of neurogenesis in the boundaries. *her4* was mainly expressed in the rhombic lip all along the AP axis and in rhombomere compartments, but was absent in the boundaries (Fig. 1F,F′,f,f′). *her9* and *her4* expression did not overlap, displaying a complementary expression pattern in the hindbrain at early stages (Fig. 1F-F″,f-f″). By 36 hpf, when Notch was active in hindbrain boundaries, most boundary cells displayed *her4* (Fig. S1C,C′,c,c′) and only a few still expressed *her9* (Fig. S1D,d). Hence, this decrease of *her9* coincides with the onset of Notch signaling in boundary cells (Hevia et al., 2022), and with the expression of *her4* and the engagement in neurogenesis. Overall, *her9* displays a dynamic expression in the hindbrain during early embryonic stages.

To explore whether sustained *her9* expression in boundaries relied on Notch signaling, we downregulated Notch activity using the gamma-secretase inhibitor LY411575. We targeted two different time intervals: (1) 18-24 hpf, when *her9* was highly enriched in hindbrain boundaries, and (2) 26-32 hpf, when *her9* expression in the boundaries decreased. Upon Notch downregulation from 18 to 24 hpf, *her9* and *her4* expression greatly decreased in the rhombomere compartments (Fig. 1G,H,g,h), consistent with both being Notch targets in these structures. However, sustained *her9* expression in the boundaries was maintained upon Notch downregulation (Fig. 1G,H,g,h), without compromising boundary identity (Fig. S1E,F,e,f). By contrast, when Notch was downregulated at later stages, both *her4* and *her9* expression greatly diminished, except for some remaining *her9* in the lateral domains (Fig. 1I,J,I,j). This coincided with the loss of expression of boundary markers (Fig. S1G,g; Letelier et al., 2018) and the onset of Notch activity in the boundaries (Hevia et al., 2022). Therefore, we conclude that the sustained *her9* expression in hindbrain boundaries is Notch independent.

### Her9 maintains the stemness of hindbrain boundary cells at early embryonic stages

**Fig. 2.**
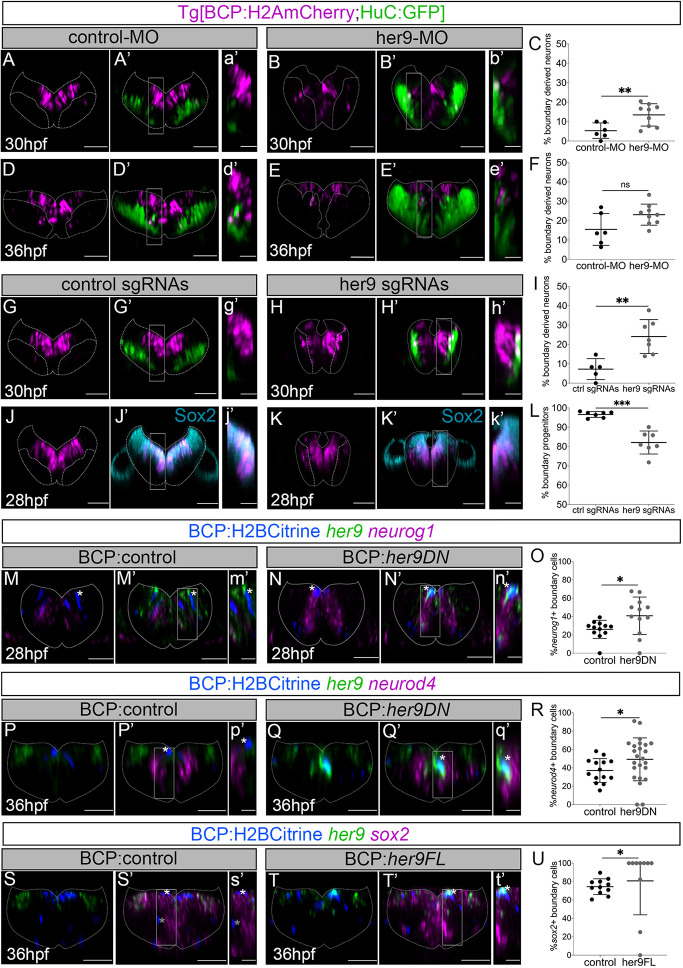
**Her9 maintains boundary cells as progenitors, preventing them from engaging in neurogenesis.** (A,B,D,E,A′,B′,D′,E′) Tg[BCP:H2AmCherry;HuC:GFP] embryos in which boundary cell nuclei are labeled with magenta and neurons in green have been injected with control-MO (A,A′,D,D′) or her9-MO (B,B′,E,E′) and analyzed at the indicated times. (C,F) Plots displaying the percentage of boundary-derived neurons (30 hpf: control-MO 5.3±4% *n*=6 versus her9-MO 13.5±5.7% *n*=9, **P=0.007; 36 hpf: control-MO 15.5±8.2% *n*=6 versus her9-MO 23.1±5.5% *n*=9, *P*=0.08). (G,H,J,K,G′,H′,J′,K′) Tg[BCP:H2AmCherry;HuC:GFP] embryos in which boundary cell nuclei are labeled with magenta and neurons in green have been injected with control sgRNAs (G,G′,J,J′) or *her9* sgRNAs (H,H′,K,K′) and analyzed at 30 hpf or immunostained with Sox2 at 28 hpf. (I,L) Plots displaying the percentage of boundary-derived neurons at 30 hpf (control sgRNAs 7.2±5.4% *n*=5 versus *her9* sgRNAs 24.1±8.8% *n*=7 embryos, **P=0.002) or boundary progenitors at 28 hpf (control sgRNAs 96.6±1.5% *n*=7 versus *her9* sgRNAs 82.1±6% *n*=7, ***P=0.0005). (M-T,M′-T′) Tg[BCP:Gal4] embryos injected with H2Bcitrine:UAS (M,M′,P,P′,S,S′), H2Bcitrine:UAS:her9DN (N,N′,Q,Q′) or H2Bcitrine:UAS:her9FL (T,T′) and *in situ* hybridized with *her9* and *neurog1* (M,N,M′,N′), *neurod4* (P,Q,P′,Q′), or *sox2* (S,T,S′,T′) probes at the indicated times. (O,R,U) Plots of the percentage of boundary cells expressing *neurog1* [control: 26.1±9.8% (of 33±17.5 cells), *n*=12 boundaries, *N*=6 embryos; her9DN: 40.8±20.4% (of 16±10.1 cells), *n*=12 boundaries, *N*=7 embryos, *P=0.038]; *neurod4* [control: 37.1±12.9% (of 29±8.7 cells), *n*=14 boundaries, *N*=4 embryos; her9DN: 49.2±23.4% (of 11±5.6 cells), *n*=24 boundaries, *N*=7 embryos; *P=0.046]; or *sox2* [control: 74.5±8.5% (of 31±11.7 cells), *n*=11 boundaries, *N*=4 embryos; her9FL: 80.7±36.9% (of 5±4.9 cells), *n*=10 boundaries, *N*=5 embryos; *P=0.026]. All plots show mean±s.d. Welch's test was performed in all comparisons except for the data shown in U, which was analyzed by Mann–Whitney. All transverse views of r4/r5 except for r3/r4 in S,T,S′,T′. (a′-t′) Magnifications of the framed regions in A′–T′. Dotted lines delimitate the contour of the neural tube. BCP, boundary cell population; hpf, hours post-fertilization; MO, morpholino; ns, not significant. Scale bars: 50 µm (main panels); 20 µm (magnifications).

To determine whether Her9 could maintain boundary cells in the progenitor state, we knocked down *her9* by injecting embryos with a splice-blocking morpholino (MO) (her9-MO; Fig. S2A) and analyzed its impact on boundary cell fate. First, we evaluated the efficiency of the her9-MO by assessing the defects in the opening of the fourth ventricle (described by Bae et al., 2005) using a cadherin reporter line (Revenu et al., 2014). her9-MO embryos showed an aberrant opening of the ventricle displaying a neural tube with a rounded shape in contrast to controls (Fig. S2B,C,b,c) that correlated with the presence of defective *her9* spliced forms (Fig. S2D). To study the role of Her9 in boundary cell fate, control-MO and her9-MO were injected into transgenic Tg[BCP:H2AmCherry;HuC:GFP] embryos, which express mCherry in the boundary cell nuclei and GFP in the differentiated neurons (Hevia et al., 2022). When neurogenesis is mostly restricted to the rhombomeres (30 hpf), we observed an increase in the percentage of boundary-derived neurons upon *her9* downregulation compared to controls (Fig. 2A-C,A′,B′,a′,b′). However, at 36 hpf, when boundary cells had already started the neurogenic program, no significant differences in boundary-derived neurons were observed (Fig. 2D-F,D′,E′,d′,e′). These results indicate that *her9* downregulation results in a premature engagement of boundary cells into neurogenesis at early stages. To reinforce these results, we decided to use a CRISPR/Cas9-based approach that redundantly targets single genes consistently producing null phenotypes in F_0_ (Hoshijima et al., 2019; Wu et al., 2018). We disrupted *her9* using four different sgRNAs (Fig. S2E) and analyzed previously described phenotypes for *her9^−/−^* mutants (Coomer et al., 2020), such as defects in the opening of the neural tube and smaller eye size (Fig. S2F-I). *her9* sgRNA-injected embryos showed an increase of boundary-derived neurons at 30 hpf, as observed in the *her9* morphants (Fig. 2G-I,G′,H′,g′,h′). Consistent with this, a reduction in boundary progenitors was observed in *her9* sgRNAs embryos compared to controls (28 hpf; Fig. 2J-L,J′,K′,j′,k′). Therefore, Her9 maintains boundary cells as progenitors, preventing them from undergoing neurogenesis.

To manipulate Her9 specifically in boundary cells, we performed conditional Her9 loss- and gain-of-function assays and analyzed the impact on the expression of cell fate genes (Fig. S2J-M). We generated embryos with boundary cells expressing either a dominant-negative form of *her9* (her9DN) or the full-length *her9* (her9FL) by injecting UAS-driven her9DN/FL constructs in Tg[BCP:Gal4] embryos, in which all cells from the boundary cell population express Gal4 after 24 hpf (Fig. S2J,K; Hevia et al., 2022). To assess the neuronal fate of boundary cells expressing her9DN, we analyzed the expression of the proneural gene *neurog1* (Guillemot, 2007) at early stages. We detected a higher percentage of her9DN boundary cells expressing *neurog1* compared to control ones (Fig. 2M-O,M′,N′,m′,n′). Accordingly, half of the her9DN cells expressed the neuronal differentiation gene *neurod4* at 36 hpf*,* a significant increase compared to control cells (Fig. 2P-R,P′,Q′,p′,q′). Thus, decreasing the function of Her9 increased the number of boundary cells committed to the neuronal lineage. Next, we determined whether Her9 was also sufficient to maintain boundary cells as progenitors by overexpressing her9FL in the boundaries for longer. We observed an increase of her9FL boundary cells in the *sox2* progenitor domain compared to control cells (Fig. 2S-U,S′,T′,s′,t′). Hence, sustaining *her9* expression keeps boundary cells in the progenitor state. Altogether, these data indicate that Her9 is necessary and sufficient to maintain the stemness of boundary cells by inhibiting the neurogenic program.

To investigate further how Her9 prevents boundary cells from entering neurogenesis, we monitored Notch activity upon disruption of *her9*. We found an increase of Notch-active boundary cells already at 30 hpf in both her9-MO and *her9* sgRNA-injected embryos compared to control ones (Fig. S3A-C,A′,B′,a′,b′,D-F,D′,E′,d′,e′). Upon analysis of the main Notch ligand expressed in the boundaries (Hevia et al., 2022), we detected a significant increase of her9DN cells expressing *deltaD* compared with control cells (28 hpf; Fig. S3G-I,G′,H′,g′,h′).These results indicate that Her9 may inhibit *deltaD* expression, and thus Notch activity, preventing the onset of neurogenesis in hindbrain boundary cells.

### Her9 controls the behavior of boundary cells

**Fig. 3.**
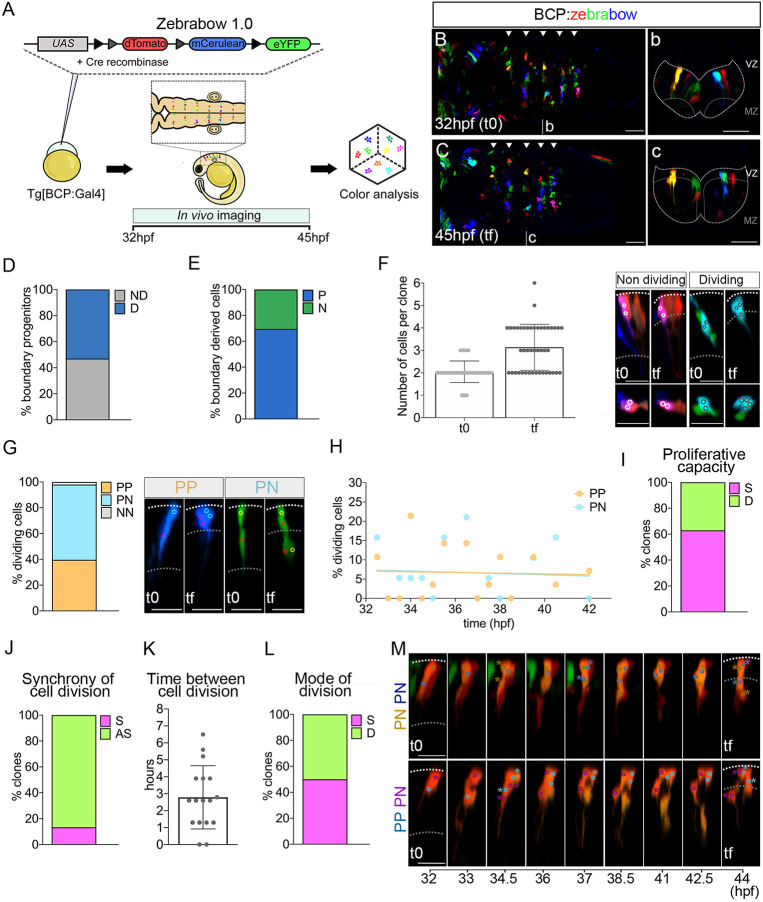
**Multicolor clonal analysis of boundary cells.** (A) Scheme depicting the experimental design to label and trace the lineage of multiple boundary progenitors. Tg[BCP:Gal4] embryos at the one-cell stage were injected with Cre protein and the UAS:zebrabow1.0 construct, which contains the fluorescent genes dTomato, mCerulean, and eYFP. Hindbrains were imaged from t0 (32 hpf) to tf (45 hpf). (B,C,b,c) Dorsal and transverse projections through r4/r5 at 32 hpf and 45 hpf. Arrowheads indicate the position of the hindbrain boundaries. Dotted line delimitates the contour of the neural tube. Scale bars: 50 μm. See Movie 1. (D) Proliferative capacity of boundary progenitors (53% dividing (D) versus 47% non-dividing (ND), *n*=90]. (E) Fate of boundary cell derivatives at 45 hpf: 70% were progenitors (P), and 30% neurons (N) (*n*=138 tracked cells). (F) Number of cells per clone at 32 hpf and 45 hpf; (t0, 2.1±0.5 cells; tf, 3.1±1 cells; *n*=44 clones). Images show examples of non-dividing (white outlined circles) and dividing (black outlined circles). (G) Graph showing the cell division modes of boundary progenitors (*n*=48 cells): symmetric proliferative (PP, 40%), asymmetric (PN, 58%), and symmetric neurogenic (NN, 2%). Images show examples of PP and PN divisions of two-cell clones. (H) Plot showing the percentage of boundary cells displaying PP (orange) or PN (turquoise) division mode over time (PP, 19 cells; PN, 28 cells; *n*=44 clones). Linear regression lines for PP [R^2^=0.002; *P*=0.9 (not significant) and for PN (R^2^=0.004; *P*=0.8 (not significant)] tend to zero. (I,J) Clonal analysis of the proliferative capacity of sister cells and their cell division synchrony, respectively. Percentage of clones in which sisters display the same (S; 63%) or different (D; 37%) proliferative behavior (*n*=44 clones); and dividing boundary clones with sister cells displaying synchronic (S; 13%) or asynchronic (AS, 87%) cell divisions (*n*=15 clones). (K) Hours between cell division of sister cells (2.8±1.9 h, *n*=17 sister cells, 15 clones). (L) Clonal analysis of the cell division mode of sister cells. Percentage of dividing boundary clones with sister cells displaying the same (S; 50%) or different (D; 50%) division mode (*n*=18 clones). (M) Examples of sister cells displaying the same (PN, PN) or different (PP, PN) division mode. In the top row, both the brown and the blue cell lineage display PN divisions, the first at 34.5 hpf and the second at 37 hpf. In the bottom row, both cells divide at 34.5 hpf, the cell from the magenta lineage undergoes a PN, and the blue one a PP division. All clone images show transverse projections with dorsal to the top. Cell centers are circled and color-coded according to their lineage. Dotted lines demarcate the ventricular zone (VZ) and the mantle zone (MZ) at 32 hpf and 45 hpf. *N*=4 embryos. Scatter plots show the mean±s.d. BCP, boundary cell population; hpf, hours post-fertilization. Scale bars: 20 µm.

Hindbrain boundary cells transition from symmetrically dividing to asymmetrically dividing progenitors relying on Notch3 activity (Hevia et al., 2022), which occurs at the time *her9* decreases in the boundaries. To unveil the role of Her9 in boundary cell behavior, we first established a system to allow us to study this transition at the single-cell and clonal level to later assess their behavior upon Her9 disruption. Thus, we performed multicolor clonal analysis using the zebrabow1.0 system (Pan et al., 2013), which enables specific boundary cells to be labeled in different colors in such a manner that clonally related cells display the same color (Fig. 3A). We generated colored boundary clones and tracked them *in vivo* from 32 (t0) to 45 (tf) hpf to reconstruct their lineage while assessing their mode of cell division (Movie 1; Fig. 3A-C,b,c; Fig. S4A-D). We combined color and spatial criteria to ascribe cell lineage and fate simultaneously (Fig. S4A; see Materials and Methods). During the analyzed temporal window, half of the progenitors divided, and the other half did not (Fig. 3D). At the end of the timelapse (45 hpf), one-third of the boundary cells had undergone neurogenesis while the rest remained as progenitors (Fig. 3E), consistent with our previous results on boundary cell lineage (Hevia et al., 2022). This multicolor analysis allowed us to observe that most clones at 32 hpf had two cells (*n*=34/44), whereas at 45 hpf they were composed of either two (*n*=16/44) or four (*n*=17/44; Fig. 3F) cells. The most frequent cases being those of two-cell clones in which none of the cells divided (*n*=12/34; Fig. 3F), or of two-cell clones in which both cells divided (*n*=13/34; Fig. 3F). Upon cell division mode analysis, we observed that progenitors mainly divided proliferative symmetrically (PP) or asymmetrically (PN), with only one single case of neurogenic symmetric division (NN) detected (Fig. 3G). No temporally restricted distribution of PP and PN divisions in boundary cells was detected (Fig. 3H) in accordance with observations of Hevia et al. (2022). Therefore, no preference for cell division mode (PP or PN) was found in terms of the number of divisions that the clone underwent or the time at which cells divided. Moreover, the zebrabow1.0 system allowed us to analyze the behavior of sister cells. Sister cells tended to have the same proliferative capacity, meaning that either both cells in the clone divided or they did not (Fig. 3I). However, most sister cells divided asynchronously (Fig. 3J), showing a delay of 2.8 h between one sister division and the other (Fig. 3K). Sister cells could also undergo the same or a different cell division mode (Fig. 3L). Thus, both sister cells could display PP or PN divisions (Fig. 3M as an example of PN, top panel) or one sister cell could make PP and the other one a PN division (Fig. 3M, lower panel). These results suggest that the clonal relationships of boundary cells influence their proliferative capacity but not their mode or time of cell division.

**Fig. 4.**
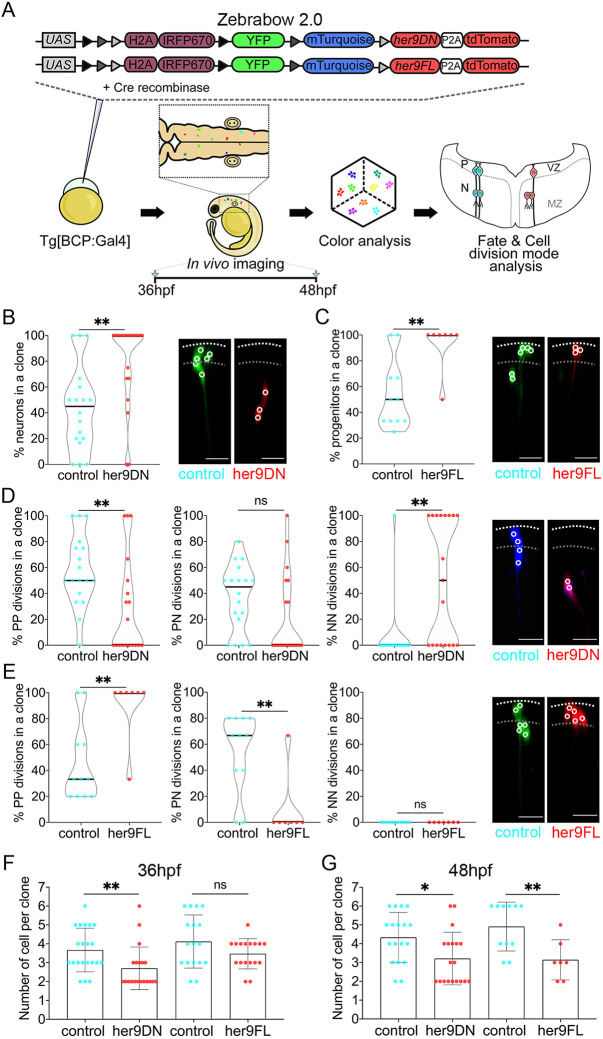
**Her9 controls the fate and behavior of boundary cells.** (A) Scheme depicting the experimental design. Tg[BCP:Gal4] embryos at the one-cell stage were injected with Cre protein and the UAS:her9DN-zebrabow2.0 or the UAS:her9FL-zebrabow2.0 constructs to conditionally modulate Her9 in the boundary cells. Transgenes contain the YFP, mTurquoise, and tdTomato genes, and tdTomato-labeled clones co-express either with her9DN or her9FL in the context of the non-red, wild-type clones. For color analysis, hindbrains were imaged at 36 hpf and 48 hpf. The clones were then analyzed for color, cell fate, and cell division mode. (B,C) Boundary cell fate upon *her9* loss (LOF) or gain (GOF) of function at 48 hpf. Violin plots show the percentage of neurons (B) or progenitors (C) in boundary control clones and upon her9DN or her9FL expression, respectively. (B) 43.4±33% of neurons in control clones (median=45%) versus 78.9±33.7% in her9DN clones (median=100%); **P=0.003. (C) 53.8±26.7% of progenitors in control clones (median=50%) versus 92.9±18.9% in her9FL clones (median=100%); **P=0.008. (D) Boundary cell division mode in *her9* LOF at 48 hpf. Violin plots show the percentage of PP (left), PN (middle), and NN (right) divisions [PP: 57.4±27.8% in control clones (median=50%), 28.6±37.7% in her9DN clones (median=0%), **P=0.007; PN: 37±25.5% in control clones (median=45%), 21.4±32.1% in her9DN clones (median=0%), *P*=0.06; NN: 5.6±23.6% in control clones (median=0%), 50±47.5% in her9DN clones (median=50%), **P=0.001]. (E) Boundary cell division mode in *her9* GOF at 48 hpf. Violin plots show the percentage of PP (left), PN (middle), and NN (right) divisions (PP: 45.5±30.7% in control clones (median=33.3%), 90.5±25.2% in her9FL clones (median=100%), **P=0.009; PN: 54.6±30.7% in control clones (median=66.7%), 9.5±25.2% in her9FL clones (median=0%), **P=0.009; NN: 0% in control clones (median=0%), 0% in her9FL clones (median=0%), *P*>0.9). Violin plots display the median in black. (F,G) Analysis of clonal cell growth upon *her9* LOF and GOF at 36 and 48 hpf. Plots show the number of cells per clone in each condition (mean±s.d.): LOF: 3.7±1.2 in control versus 2.7±1.1 in her9DN clones at 36 hpf, **P=0.003; 4.3±1.3 in control versus 3.2±1.4 in her9DN clones at 48 hpf, *P=0.016. GOF: 4.1±1.4 in control versus 3.5±0.8 in her9FL clones at 36 hpf, *P*=0.187; 4.9±1.3 in control versus 3.1±1.1 in her9FL clones at 48 hpf, **P=0.008. 36 hpf: *n*=21 control clones, *n*=20 her9DN clones (12 embryos); 48 hpf: *n*=18 control clones, *n*=19 her9DN clones (15 embryos). 36 hpf: *n*=17 control clones, *n*=17 her9FL clones (13 embryos); 48 hpf: *n*=11 control clones, *n*=7 her9FL clones (15 embryos). Mann–Whitney test was performed in all comparisons. Images show transverse projections of control, her9DN, or her9FL boundary clones with dorsal to the top. Dotted lines demarcate the ventricular zone (VZ) and the mantle zone (MZ) as depicted in A. White circles in the images indicate cell centers. BCP, boundary cell population; hpf, hours post-fertilization; PP, progenitor-progenitor division; PN, progenitor-neuron division; NN, neuron-neuron division; ns, not significant. Scale bars: 20 µm.

Next, to assess whether Her9 controls the proliferative behavior of boundary cells, we combined functional assays with multicolor clonal analysis (Loulier et al., 2014). For this, we generated a new version of the zebrabow transgenes, zebrabow2.0, in which either the her9DN or the her9FL co-expressed with one of the color labels (red). The zebrabow2.0 was under the control of UAS, allowing us to express the her9DN or the her9FL specifically in boundary cells by injecting Tg[BCP:Gal4] embryos (Hevia et al., 2022). This allowed us to modulate Her9 specifically in boundary clones identified by a specific color marker (in this case tdTomato) and compare their fate to the wild-type clones marked with distinct colors (non-red) within the same embryo (Fig. 4A). In both loss- and gain-of-function experiments, we observed high color diversity (Fig. S4E,F) and classified red and non-red clones in the same embryo at both 36 and 48 hpf. When cell fate was assessed at 48 hpf (Fig. 4A; Fig. S4A; see Materials and Methods), her9DN clones showed a significantly higher proportion of neurons whereas her9FL clones displayed more progenitors compared to control clones (Fig. 4B,C). Cell fate changes in her9DN and her9FL clones were coupled with changes in cell morphology, i.e. loss of the apical contact to the ventricle or maintenance of the apical contact and radial glial projection, respectively (Fig. 4B,C, clone images). These results reinforce the previous observation that Her9 promotes maintenance of the boundary progenitor pool.

Next, we studied whether Her9 maintains the pool of boundary progenitors by modifying their division mode. Boundary her9DN clones at 48 hpf showed a decrease in the percentage of PP divisions and an increase of NN divisions compared with control clones (Fig. 4D), whereas sustained expression of *her9* resulted in an increase of PP and a decrease in PN divisions compared to controls (Fig. 4E). These observations suggested that Her9 expands the boundary progenitor pool by maintaining PP divisions and preventing boundary cells from undergoing neurogenic divisions (PN or NN). Accordingly, when we analyzed clonal growth, we observed that already at 36 hpf the her9DN clones were smaller compared to the control ones (Fig. 4F), and this difference was maintained at 48 hpf (Fig. 4G). These results indicate that Her9 is necessary for the clonal growth of boundary cells. However, the size of control and her9FL clones at 36 hpf was similar (Fig. 4F). At 48 hpf, her9FL clones displayed fewer boundary cells than controls (Fig. 4G), indicating that her9FL clones did not grow during this temporal window. Therefore, sustained *her9* expression at later stages does not increase the growth of boundary clones. This may suggest that boundary cells could only undergo a limited number of rounds of PP cell divisions before the onset of neurogenesis.

### Her9 promotes the proliferation of boundary cells through *cdkn1ca* and Cyclin D1

**Fig. 5.**
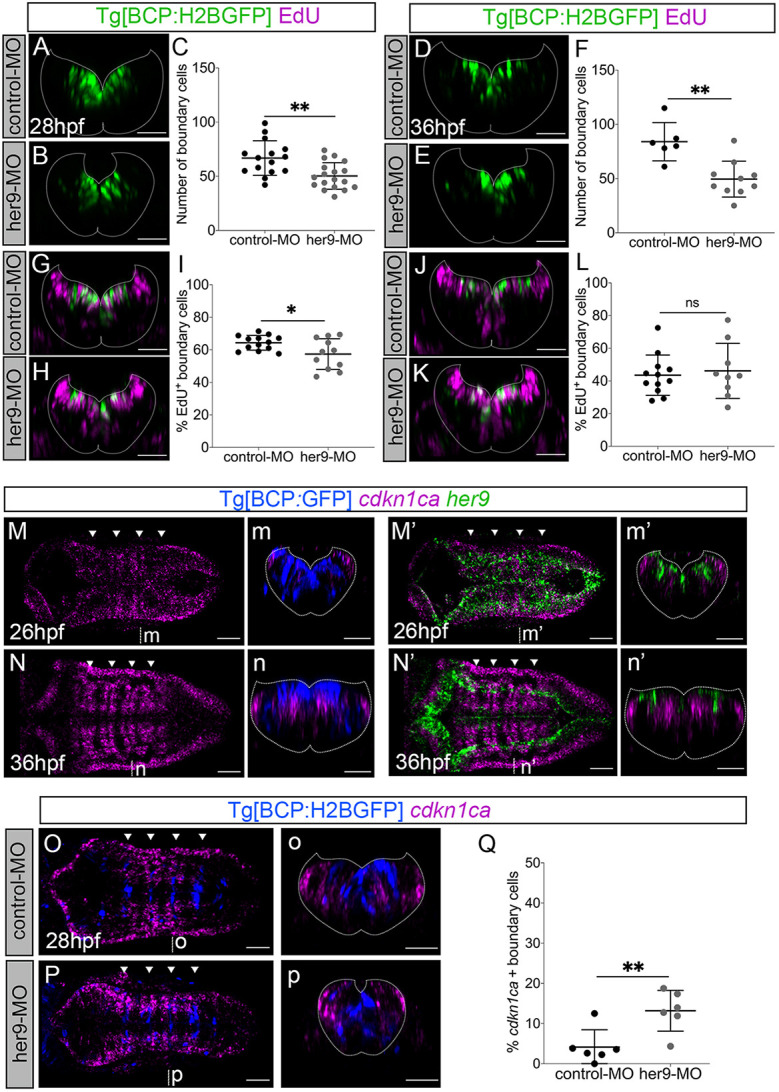
**Her9 controls boundary cell proliferation through *cdkn1ca*.** (A,B,D,E,G,H,J,K) Transverse views of Tg[BCP:H2BGFP] embryos injected with control-MO and her9-MO, displaying boundary nuclei and EdU incorporation to detect S-phase cells (G,H,J,K) at the indicated stages. (C,F) Plots displaying the total number of boundary cells in r4/r5 (28 hpf: control-MO 66.8±16 *n*=15 versus her9-MO 50.28±12.3 *n*=18, **P=0.0029, Welch's test; 36 hpf: control-MO 84±17.6 *n*=6 versus her9-MO 49.5±16.5 *n*=10, **P=0.003, Welch's test). (I,L) Plots showing the percentage of S-phase boundary cells in r4/r5 (28 hpf: control-MO 64.3±4.5% *n*=13 versus her9-MO 57.4±9.5% *n*=11, *P=0.042, Welch's test; 36 hpf: control-MO 43.5±12.3% *n*=12 versus her9-MO 46.17±16.9% *n*=9, *P*=0.695, Welch's test). (M,N,M′,N′) Dorsal views of Tg[BCP:GFP] embryos at the indicated stages *in situ* hybridized with *cdkn1ca* and *her9* probes. (O,P) Dorsal views of Tg[BCP:H2BGFP] embryos injected with control-MO or her9-MO and *in situ* hybridized with *cdkn1ca* probe at 28 hpf. (Q) Plot displaying the percentage of boundary cells expressing *cdkn1ca* (4.1±4.3% in control-MO *n*=6 versus 13.2±5.1% in her9-MO *n*=6, **P=0.009, Mann–Whitney test). (m-p,m′,n′) Transverse views of dorsal views displayed in M-P,M′,N′. The plots show mean±s.d. Dotted lines delimitate the contour of the neural tube. Arrowheads indicate the position of the hindbrain boundaries. BCP, boundary cell population; hpf, hours post-fertilization; MO, morpholino; ns, not significant. Scale bars: 50 µm.

To examine whether Her9 regulates the proliferative capacity of boundary cells, we assessed the impact of *her9* downregulation in boundary cell proliferation, when *her9* expression was enriched in boundaries (28 hpf) and at the time its expression had decreased (36 hpf). We observed fewer boundary cells upon *her9* downregulation both at 28 hpf and 36 hpf (Fig. 5A-F). To determine whether this was caused by a decrease in the number of cells entering S phase, we measured ethynyl-2'-deoxyuridine (EdU)-incorporation after a 1-h pulse and detected a lower percentage of cells in S phase in her9-MO compared to controls at 28 hpf (Fig. 5G-I), but we found no differences at 36 hpf (Fig. 5J-L). These data indicate that Her9 controls the proliferative capacity of boundary cells at early embryonic stages.

Hes/Her factors regulate the expression of cell cycle genes in different systems (Georgia et al., 2006; Maeda et al., 2023; Monahan et al., 2009; Radosevic et al., 2011; Zalc et al., 2014). To determine whether Her9 in boundary cells controls the proliferative capacity by regulating cell cycle genes, we first addressed the expression of *cdkn1ca*, a cell cycle arrest gene previously showed to be expressed in the rhombomere compartments (Amoyel et al., 2005). *cdkn1ca* was expressed in the boundary flanking regions actively engaged in neurogenesis, and it was absent in the boundaries when *her9* was highly expressed there (Fig. 5M,N,M′,N′,m,n,m′,n′). Although boundaries were devoid of *cdkn1ca* expression at early stages, some boundary cells started to express it after the loss of *her9* (Fig. 5m,n,m′,n′). These results indicate that the onset of *cdkn1ca* expression coincides with the decline of *her9* in the boundaries and their commitment to neurogenesis. To seek whether Her9 controls cell proliferation through the regulation of *cdkn1ca*, we assessed its expression upon *her9* downregulation. We observed an increase in boundary cells expressing *cdkn1ca* in her9-MO embryos compared to controls (Fig. 5O-Q,o,p). Hence, these results suggest that when Her9 is highly expressed in boundary cells it directly or indirectly represses *cdkn1ca* expression and thus promotes cell proliferation.

**Fig. 6.**
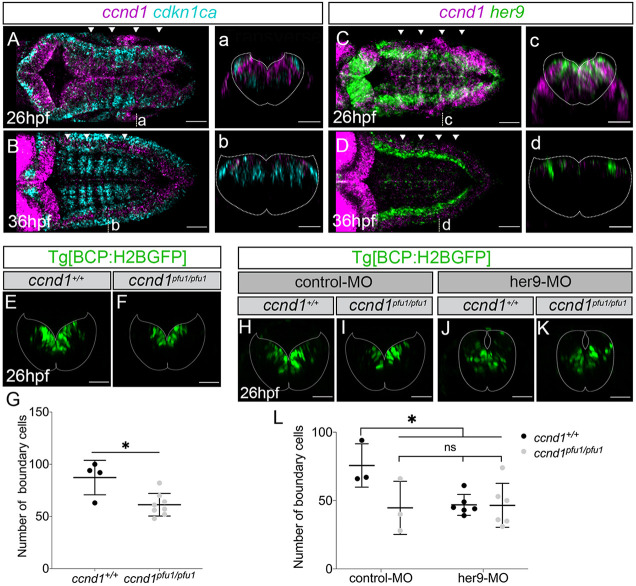
**Cyclin D1 and Her9 control the proliferation of boundary cells.** (A-D) Tg[BCP:GFP] embryos at the indicated stages were *in situ* hybridized with *ccnd1* and *cdkn1ca* or *her9* probes. (E,F) Tg[BCP:H2BGFP;*ccnd1^+/+^*] and Tg[BCP:H2BGFP;*ccnd1^pfu1/pfu1^*] embryos displaying boundary cells in green at 26 hpf . (G) Plot showing the number of boundary cells (87.3±16.5 in *ccnd1^+/+^ n*=4 versus 61.3±10.8 in *ccnd1^pfu1/pfu1^ n*=8; *P=0.04, Welch's test). (H-K) Tg[BCP:H2BGFP;*ccnd1^+/+^*] and Tg[BCP:H2BGFP;*ccnd1^pfu1/pfu1^*] embryos injected with control-MO (H,I) or her9-MO (J,K). (L) Plot showing the number of boundary cells in Tg[BCP:H2BGFP;*ccnd1^+/+^*] and Tg[BCP:H2BGFP;*ccnd1^pfu1/pfu1^*] embryos upon different conditions. (H) 75.7±15.9 in *ccnd1^+/+^* control-MO *n*=3; (I) 44.7±19.4 in *ccnd1^pfu1/pfu1^* control-MO *n*=3; (J) 46.8±7.7 in *ccnd1^+/+^* her9-MO *n*=6; (K) 46.5±16.1 in *ccnd1^pfu1/pfu1^* her9-MO *n*=6. H versus I, *P=0.04; H versus J, *P=0.03; H versus K, *P=0.03; I versus J, *P*=0.99; I versus K, *P*=0.99; J versus K, *P*>0.99. One-way ANOVA, Dunnett's multiple comparison test. Images are transverse views of the r4/r5 boundary (E,F,H-K,a-d) or dorsal maximum intensity projections of the hindbrain with anterior to the left (A-D). The plots show mean±s.d. Dotted lines delimitate the contour of the neural tube. Arrowheads indicate the position of the hindbrain boundaries. BCP, boundary cell population; hpf, hours post-fertilization; MO, morpholino; ns, not significant. Scale bars: 50 µm.

Cell cycle arrest proteins, such as the Cdkn1ca orthologous p57, regulate cell proliferation through the inhibition of Cyclin/Cdk complexes (Grison and Atanasoski, 2020). Thus, we next studied the dynamics of the cell cycle progression Cyclin D1 gene (*ccnd1*), which is expressed in hindbrain boundaries (Amoyel et al., 2005). *ccnd1* was enriched in hindbrain boundaries at early embryonic stages, but greatly decreased in the whole hindbrain by 36 hpf (Fig. 6A,B,a,b). *ccnd1* did not overlap with *cdkn1ca* (Fig. 6A,B,a,b), whereas it did with *her9* in boundary cells at 26 hpf and their expression in the boundaries concomitantly decreased by 36 hpf (Fig. 6C,D,c,d; Fig. S5A-A″,a-a″). Therefore, *ccnd1* and *cdkn1ca* displayed complementary spatiotemporal patterns, whereas *ccnd1* and *her9* overlapped in the hindbrain boundaries. To explore the putative role of Cyclin D1 in the proliferation of boundary cells, we generated a *ccnd1* loss-of-function mutant (Fig. S5B) and analyzed its effects. A significant reduction in the number of boundary cells was observed in *ccnd1^pfu1/pfu1^* mutants compared to *ccnd1^+/+^* embryos (Fig. 6E-G), similar to that observed in *her9* morphants (Fig. 5C, Fig. 6G). To examine whether Cyclin D1 is modulated by Her9, we injected her9-MO into *ccnd1^+/+^* embryos and *ccdn1^pfu1/pfu1^* mutants and analyzed the effect on boundary cell number. No additive effects were detected in the decrease of the number of boundary cells between control and *her9* downregulated *ccdn1^pfu1/pfu1^* mutants compared to *ccnd1^+/+^* embryos (Fig. 6H-L). Thus, Cyclin D1 impacts the proliferative capacity of boundary cells potentially downstream of Her9. Overall, we propose that Her9 could control Cyclin D1 activity in the boundaries through the repression of *cdkn1ca*, and thus promote proliferation in boundary cells.

### *her9* is enriched in radial glial progenitors in a Notch-independent manner at late embryonic stages

**Fig. 7.**
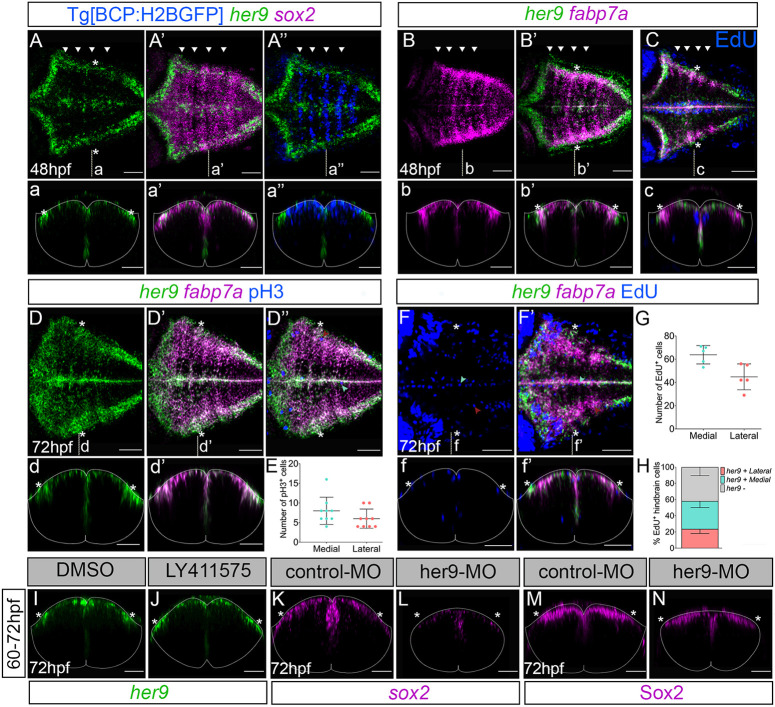
***her9* is expressed in hindbrain progenitor populations at late embryonic stages.** (A-A″,B-B″) Tg[BCP:H2BGFP] *in situ* hybridized with *her9* and *sox2* (A′) or *fabp7a* (B,B′) probes at the indicated stages*.* Arrowheads indicate the position of the hindbrain boundaries. (D-D″) Embryos at 72 hpf hybridized with *her9* and *fabp7a* probes and immunostained with anti-pH3. (C,F-F′) Tg[BCP:H2BGFP] embryos at the indicated stages assayed for EdU incorporation to detect cells in S phase (*n*=6/6 at 48 hpf and *n*=5/5 at 72 hpf). Turquoise and red arrowheads indicate the position of mitotic or proliferating cells in lateral and medial domains, respectively. (A-F,A′-F′,A″,D″) Dorsal maximum intensity projections of the hindbrain with anterior to the left. (E,G) Plots showing the number of mitotic cells or S-phase cells in *her9*-expressing domains (E: 8±3.5 cells in the medial versus 6±2.5 cells in the lateral domain, *n*=9; G: 63.8±7.9 cells in the medial versus 44.8±11.1 cells in the lateral domain, *n*=5]. (H) Plot showing the percentage of S-phase cells in hindbrain domains (23.7±5.6% in the *her9*-expressing lateral versus 34.5±8% in *her9*-expressing medial versus 41.9±10.5% in the *her9*-negative domain, *n*=5). (I,J) Embryos treated with DMSO or LY411575 from 60 hpf to 72 hpf, and *in situ* hybridized with *her9* probe (DMSO *n*=7/7; LY411575 *n*=6/6). (K-N) Embryos injected with control-MO and her9-MO, and *in situ* hybridized with *sox2* probe or immunostained with anti-Sox2 at 72 hpf. Note that *sox2* expression domain (K-M) was decreased in the her9-MO embryo (L, *n*=6/7; N, *n*=8/9) compared to the control (K, *n*=7/7; M, *n*=8/8). (a-a″,b-b′,c,d-d′,f-f′,I-N) Transverse projections of r4/r5 boundary. Asterisks indicate the position of the *her9*-expressing lateral domains. Dotted line delimitates the contour of the neural tube. BCP, boundary cell population; hpf, hours post-fertilization; MO, morpholino. Scale bars: 50 µm.

*her9* expression was temporarily sustained in hindbrain boundaries at early stages. At later stages, *her9* was enriched in other hindbrain territories, including two lateral domains along the AP axis (Fig. 1E; Fig. S1D). To explore this *her9* cell population further, we profiled it for the expression of the neural progenitor and radial glia markers *sox2* and *fabp7a*, respectively. At 48 hpf, *her9* was mainly expressed in the most medial and lateral hindbrain territories, where it colocalized with *sox2* along the ventricular domain, with few boundary cells still expressing it (Fig. 7A-A″,a-a″, asterisks)*. fabp7a* colocalized with *her9* in most of these medial and lateral domains both at 48 hpf and 72 hpf (Fig. 7B-D,B′,D′,b-d,b′,d′). We detected mitotic events in the *her9* lateral and medial populations (Fig. 7D″,E). Accordingly, we observed *her9*-expressing radial glial progenitors entering S phase in both domains, although this mainly occurred in the medial part at 48 and 72 hpf (Fig. 7C,F,F′,G,H,c,f,f′, turquoise and red arrowheads). However, few cells in the hindbrain proliferated at 72 hpf, suggesting that the remaining *her9*-positive progenitors were mostly slow dividing or quiescent cells. Overall, *her9* is highly enriched in radial glia progenitors at the time neurogenesis and cell proliferation decreases substantially in the hindbrain. Next, we assessed whether this late *her9* expression depended on Notch activity. Upon Notch downregulation, we observed no differences in *her9* expression in these domains (Fig. 7I,J). When we assessed the impact of *her9* downregulation, we detected a strong decrease in *sox2* expression and shrinking of the Sox2-protein domain in the whole hindbrain, including the lateral domains (Fig. 7K-N). Taken together, our results suggest that Her9 plays a role in maintaining the stemness of distinct hindbrain progenitors’ populations in a Notch-independent manner at different temporal windows.

## DISCUSSION

**Fig. 8.**
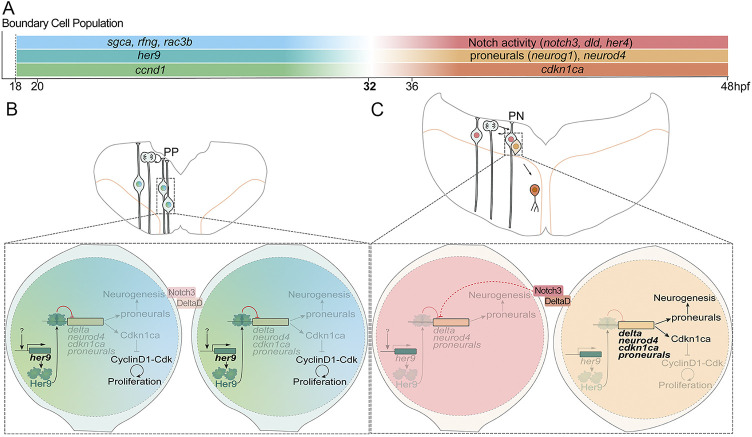
**The strategy of stemness maintenance and expansion of hindbrain boundary cells.** (A) Scheme of temporal gene expression in the boundary cell population before and after 32 hpf. (B,C) Scheme depicting transverse representations of the hindbrain boundaries before and after 32 hpf. Magnifications of cells (bottom) displaying the mechanisms of Her9 action. Before 32 hpf (B), Her9 inhibits neurogenic genes and cell cycle genes, such as *cdkn1ca*, keeping boundary cells as progenitors dividing symmetric proliferative. Upon *her9* decline (C), Notch3/DeltaD trigger asymmetric divisions. One cell is kept as a progenitor through Notch3 activity, whereas the other cell expresses both neurogenic genes and *cdkn1ca*, promoting neurogenesis and cell cycle exit.

*her9* is highly expressed in boundary cells at the time these cells are specified, thus coinciding with the expression of the boundary genes (Fig. 8A; (Cheng et al., 2004; Letelier et al., 2018). Her9 maintains the stemness of boundary cells when they display their crucial functions as mechanical barriers and signaling centers (Calzolari et al., 2014; Riley et al., 2004; Terriente et al., 2012). It represses the boundaries’ neurogenic program by inhibiting *neurog1* and *neurod4* (Fig. 8B), consistent with the role of Hes1 in the murine brain (Baek et al., 2006; Hatakeyama et al., 2004; Hirata et al., 2001). In the absence of Notch activity, Her9 expands the pool of boundary progenitors by promoting symmetric proliferative divisions and preventing neurogenic divisions (Fig. 8B). Later, *her9* loss coincides with the onset of Notch3 signaling and *her4* expression, promoting the transition of boundary cells to radial glia progenitors undergoing asymmetric divisions (Fig. 8A,C). These results provide insights into the model in which Hes genes maintain the stemness of neural progenitors before the onset of Notch activity (Hatakeyama and Kageyama, 2006). her9DN clones show an increase of symmetric neurogenic cell divisions at the expense of symmetric proliferative cell divisions as observed in compound Hes mouse mutants (Hatakeyama et al., 2004). However, the moderate neuronal differentiation and increased Notch activity of boundary cells upon *her9* loss of function suggest that Her9 could also prevent the onset of asymmetric cell divisions in hindbrain boundaries by repressing Notch activity through the inhibition of *deltaD* expression (Fig. 8B,C). Our data are in line with those of Hevia et al. (2022), indicating that the boundary cell division mode is not prefigured by time or space. We showed that the mode of division it is also not determined by clonal relationships, pointing towards a more stochastic model of cell behavior after the onset of neurogenesis, similar to that of progenitor cells in the teleost retina (He et al., 2012). Nevertheless, the proliferative capacity of boundary cells seems to be deterministic, as clone size shows little variability and sister cells tend to behave similarly in terms of proliferation even if their time of division is not synchronized.

Furthermore, Her9 promotes boundary cell proliferation by potentially repressing *cdkn1ca* expression at early embryonic stages. In contrast, high levels of Hes1 promote low cell proliferation or quiescence (Baek et al., 2006; Shimojo et al., 2008; Sueda et al., 2019). Consistent with this, boundary cells are highly proliferative in teleosts as opposed to amniotes (Baek et al., 2006; Peretz et al., 2016; Voltes et al., 2019). This functional difference between high Her9 and high Hes1 could be explained by a newly described mechanism whereby high levels of Hes1 activates or inhibits proliferation of neural stem cells according to its shorter or longer time of expression, resulting in the repression or activation of the cell cycle arrest protein p21, respectively (Maeda et al., 2023). The enrichment of *ccnd1* in hindbrain boundaries and its effects on the number of boundary cells, suggest that Cyclin D1 controls their proliferative capacity (Fig. 8A,B). We propose a model in which, upon the inhibition of *cdkn1c*a by Her9, Cdkn1ca repression of Cyclin D1 activity is reduced, promoting a faster cell cycle progression of boundary cells compared to the neighboring rhombomeric cells at early stages (Fig. 8B). Thus, when Her9 decreases, boundary cells would express Cdkn1ca and exit the cell cycle (Fig. 8C). Boundary cell proliferation is also controlled by Yap/Taz-TEAD activity downstream of actomyosin contraction (Voltes et al., 2019). Hence, Her9 and Tap/Taz-TEAD could cooperate or act upstream of each other to synergistically promote cell proliferation by regulating cell cycle-related genes (Engel-Pizcueta and Pujades, 2021; Mamidi et al., 2018). Future functional studies should be performed to address their interplay and the regulation of the cell cycle in boundary cells.

*her9* expression is highly dynamic during hindbrain development. Early in development, its expression is temporarily enriched in boundary cells. Later, it remains mainly in radial glial cells of lateral domains all along the AP axis. Our results support the developmental strategy whereby different Hes/Her genes maintain distinct neural progenitor populations (Chapouton et al., 2006; Sigloch et al., 2023; Stigloher et al., 2008), establishing the neurogenic asynchrony in neural tissues. Hindbrain boundaries initially behave as a pool of non-neurogenic progenitors expressing *her9* independently of Notch. Later, they express *her4* and behave as a classical proneural cluster, as do progenitors in telencephalic roof plate (Dirian et al., 2014). Like the boundaries, the remaining hindbrain progenitors at later stages express *her9* independently of Notch. Accordingly, Sox2 protein is enriched in the lateral domains when Notch activity ceases in the hindbrain (Hevia et al., 2022), and these progenitors are reduced upon *her9* downregulation. Thus, we propose Her9 as a common player for maintaining distinct progenitor populations in the hindbrain at different temporal windows in a Notch-independent manner. Non-oscillatory Hes1 expression correlates with high Hes1 promoting the stemness and low proliferation in neural progenitors (Baek et al., 2006; Shimojo et al., 2008; Sueda et al., 2019). Whether Her9 oscillatory dynamics could explain the differences between distinct hindbrain progenitor populations in space and time is still an open question. Notably, derived mid-hindbrain boundary *her5* cells and telencephalic roof plate *her9/her6* progenitors show delayed neurogenesis and constitute a reservoir of neural stem cells in the adult brain (Chapouton et al., 2006; Dirian et al., 2014). Since very few boundary progenitors remain at later stages (Hevia et al., 2022), it seems unlikely that they would persist until adulthood. However, *her9*-positive domains of the hindbrain containing slow dividing or quiescent radial glial cells could harbor long-lasting progenitors until the adult stages. Future long-term lineage tracing of *her9* progenitors would be crucial to test this hypothesis.

## MATERIALS AND METHODS

### Ethics declarations and approval for animal experiments

All procedures were approved by institutional animal care guidelines, the PRBB Ethics Committee in Animal Experimentation and the Departament de Territori i Sostenibilitat (Generalitat of Catalonia) in compliance with the National and European regulations. The PRBB animal facility has the AAALAC International approval B9900073. All the members accessing the animal house must hold the international FELASA accreditation. The Project License covering the proposed work (Ref. 10642, GC) pays particular attention to the 3Rs.

### Zebrafish strains

All zebrafish strains were maintained alternating generations of in-crosses and out-crosses with wild type. Embryos were obtained by mating adult fish following standard methods and grown at 28.5°C or 23°C. 1-Phenyl-2-thiourea (PTU) (1%; Sigma-Aldrich) was used as an inhibitor of pigmentation from 24 hpf onward. Tg[BCP:Gal4], Tg[BCP:H2AmCherry] pfu102Tg, Tg[BCP:GFP], and Tg[BCP:H2BGFP] pfu103Tg lines label hindbrain boundary cells (Hevia et al., 2022). Tg[elA:GFP] ens1Tg and Mü4127 zf278Et were used as landmarks of r3 and r5 (Distel et al., 2009; Labalette et al., 2011). The Tg[HuC:GFP] knu3Tg line labels differentiated neurons (Park et al., 2000). The Tg[tp1:d2GFP] mw43Tg line monitors Notch activity (Hevia et al., 2022; Park et al., 2000). The Tg[cdh2:GFP] zf518Tg line corresponds to the tandem fluorescent cadherin timer, which allows *in vivo* monitoring of cadherin2 subcellular location (Revenu et al., 2014).

### MO knockdown experiments

Embryos were injected at the one-cell stage with: (1) 8 ng random 25N MO as control (Gene Tools, LLC); or (2) 8 ng splicing-blocking her9-2MO (Fig. S2A, her9-MO in the text; Bae et al., 2005). All MO injections also included 7.5 ng p53-MO (Langheinrich et al., 2002) to reduce putative artifacts (Gerety and Wilkinson, 2011). To assess her9-MO efficiency, we analyzed fourth ventricle opening defects (Bae et al., 2005). The penetrance of this phenotype was around 80% (control-MO *n*=0/8 versus her9-MO *n*=10/13 embryos; Fig. S2B,C,b,c), although some degree of variability was observed between independent experiments. The presence of the *her9* spliced-defective variants was checked in the same control-MO and her9-MO embryos at 36 hpf (Fig. S2D). RNA of the two pools of embryos was extracted with TRIzol reagent (Ambion) and phenol/chloroform protocol. RT-PCR was performed with the SuperScript III Kit (Invitrogen) following the manufacturer's instructions. The MO-binding site was amplified using the primers described in Table S1.

### CRISPR/Cas9-based approach to target *her9* in F_0_

Four different CRISPR single RNAs guides (sgRNAs) were used to target *her9* redundantly (Fig. S2E) following previously published guidelines (Hoshijima et al., 2019; Wu et al., 2018). We targeted the ATG start of the gene (#1), and the main functional domains: (1) the beginning of the dimerization HLH domain (exon2, #2); (2) the orange domain required for heterodimerization selection (exon4, #3) (Taelman et al., 2004); and (3) before the transcriptional repressor WRPW domain (exon4, #4) (Fisher et al., 1996; McLarren et al., 2001) (Fig. S2E). *her9* sgRNAs were designed with the CHOPCHOP platform (https://chopchop.cbu.uib.no/) and following previously published guidelines (Wu et al., 2018). As negative controls, we used control sgRNAs not targeting the zebrafish genome designed by randomization of antibiotic resistance gene sequences. All CRISPR RNAs (crRNAs) were ordered from Integrated DNA Technologies (IDT) (Table S1). Each crRNA was duplexed with Alt-R™ tracRNA (1:1, IDT). Embryos were injected at the one-cell stage with a combination of the four *her9* or control duplex gRNAs at 20 µM (5 µM each dgRNA) and Alt-R™ S.p. HiFi Cas9 Nuclease v3 (IDT) at 6.2 µM in a 1 nl drop. Then, embryos were either *in vivo* imaged or fixed for immunostaining. To assess the *her9* sgRNA efficiency, we analyzed the previously reported eye size defects in *her9^−/−^* embryos (Coomer et al., 2020) and opening of the fourth ventricle defects in *her9* morphants (Fig. S2B,C,F-I,b,c).

### TALEN genome editing

The *ccnd1^pfu1^* line was generated using TALEN-induced mutagenesis. A target site in the second exon and the corresponding left and right TALENs were designed using the online software MoJo Hand (http://www.talendesign.org). The TALEN repeat arrays were generated following the protocol described by Cermak et al. (2011), and are available on the Addgene website (https://www.addgene.org/talen/guide/). The array plasmids were fused to Fok1 endonuclease and linearized. mRNAs were *in vitro* transcribed using the T3 mMessage mMachine Kit (Ambion). The mRNAs of the left and right arms (1:1) were injected into one-cell-stage embryos. gDNA of a subset of embryos from each clutch was extracted. The efficiency of the TALEN pair was assessed by amplifying a 550 bp PCR fragment containing the target sites (Table S1) and then digesting with BfuI, which make a cut in the spacer of the target site (Fig. S5C). Mosaic fish were out-crossed and a pool of embryos was genotyped as described to identify potential mutations. F1 fish were genotyped using fin clips and the PCR band carrying the mutation was sequenced. The *ccnd1^pfu1^* mutation consisted of a deletion of two nucleotides at positions c.35 to c.36 replaced by T (Fig. S5B). This change causes a premature STOP codon generating a truncated Cyclin D1 protein of 12 amino acids without the cyclin box domain. To study the effects of the mutation in the boundary cells, *ccnd1^pfu1/+^* were out-crossed with Tg[BCP:H2BGFP] fish, and the progeny was raised to adulthood. Sibling embryos obtained from Tg[BCP:H2BGFP;*ccnd1^pfu1/+^*] out-crossed with *ccnd1^pfu1/+^* fish were *in vivo* imaged and genotyped. For the *her9* and *ccnd1* epistasis experiment, sibling embryos from Tg[BCP:H2BGFP;*ccnd1^pfu1/+^*] out-crossed with *ccnd1^pfu1/+^* were injected with the control-MO and her9-MO at the one-cell stage as previously described and *in vivo* imaged at the desired stages. Genotyping was performed after the imaging.

### Pharmacological treatments

Embryos were dechorionated and treated with either 10 μM of the γ-secretase inhibitor LY411575 (Sigma-Aldrich, SML0506-5MG) as an inhibitor of Notch signaling, or DMSO (CHEM-LAB, CL00.0422) as a control, diluted in embryo medium. Embryos were incubated during the indicated temporal windows at 28.5°C. After treatment, embryos were washed with embryo media and fixed in 4% paraformaldehyde (PFA) for 3 h at room temperature or overnight at 4°C.

### Whole-mount *in situ* hybridization

Embryo whole-mount *in situ* hybridization was adapted from Thisse and Thisse (2008). The following antisense riboprobes were generated by *in vitro* transcription from cloned cDNAs: *deltaD* (Haddon et al., 1998), *her9* (Leve et al., 2001), *neurod4* (Park et al., 2003), *neurog1* (Itoh and Chitnis, 2001), *sox2* (März et al., 2010), and *sgca* and *rfng* (Letelier et al., 2018). The other antisense probes were generated by PCR amplification adding the promoter sequence T7 or Sp6 in the reverse primers (Table S1). Embryos were dehydrated and posteriorly rehydrated before permeabilization with proteinase K (10 mg/ml, Invitrogen) within a range of 5-30 min according to the stage (18-72 hpf). They were incubated in FLUO- (1:50) and DIG- (1:100) labeled probes diluted in hybridization buffer. After washings, they were incubated with an anti-FLUO-POD (1:400; Roche, 11426346910) in 2% blocking reagent (Roche), 10% neutralized goat serum in 1× malic acid buffer in PBT (MABT) blocking solution, followed by anti-DIG-POD (1:400; Roche, 11207733910). FLUO- and DIG-labeled probes were revealed with TSA Fluorescein and Cy3 (Akoya, NEL753001KT), respectively.

### *In toto* embryo immunostaining

Embryos were permeabilized with proteinase K (10 mg/ml, Invitrogen) in 1% PBS, 0.1% Tween20 (PBT), post-fixed with 4% PFA, and blocked in 10% NGS and 2% bovine serum albumin in PBT for 2 h at room temperature. For immunostaining after *in situ* hybridization, embryos were blocked in 5% neutralized goat serum in PBT for 1 h. Embryos were incubated overnight at 4°C with rabbit anti-GFP (1:400; Torrey Pines, TP401), rabbit anti-pH3 (1:200; Upstate, 06-570), or mouse anti-HuC (1:400; Thermo Fisher Scientific, A-21271). Sox2 staining was performed using mouse anti-Sox2 (1:200; Abcam, ab171380) following a previously published protocol (Sigloch et al., 2023). After washings with PBT, embryos were incubated with secondary antibodies conjugated with Alexa Fluor^®^ 488, 594 or 633 (1:500; Invitrogen, A-11029, A-21206, A-11037, A-21070 or A-21053). DRAQ5 (1:2000; Biostatus, DR50200) and Alexa Fluor™ 568 Phalloidin (1:500; Invitrogen, A12380) in 5% DMSO were used to stain nuclei and cell membranes, respectively.

### EdU incorporation experiments

Cells in S phase were detected by EdU incorporation using the Click-It™ EdU Alexa Fluor™ 647 Imaging Kit (Thermo Fisher Scientific, C10340) according to Belmonte-Mateos et al. (2023). Briefly, Tg[BCP:H2BGFP] embryos were dechorionated, incubated in 500 µM EdU diluted in 7% DMSO fish water for 1 h with shaking on ice for better EdU incorporation. They were washed with embryo medium and fixed in 4% PFA overnight at 4°C. Embryos were permeabilized with proteinase K (10 mg/ml, Invitrogen), post-fixed, and washed in PBT. Then, they were incubated for 1 h in 1% DMSO/1% Triton X-100/PBS. The Click-iT reaction was carried out according to the manufacturer's instructions before immunostaining or *in situ* hybridization.

### Confocal imaging of whole-mount embryos

Embryos were mounted in either 1% low melting point agarose (Ecogen) or 0.7% for time-lapse imaging, with the hindbrain towards the glass of the glass-bottom Petri dishes. Low melting point agarose was dissolved in embryo water with 1% PTU (Sigma-Aldrich) or PBT for live and fixed embryos, respectively. Live embryos were anesthetized with 0.1% tricaine (Sigma-Aldrich). All images were acquired using an SP8 Leica inverted confocal microscope. Different combinations of 458, 488, 514, 561, and 633 nm lasers were used to excite fluorochromes and emitted light was detected with PMT or HyD detectors. Each channel was acquired by line in live embryos or by stacks in fixed embryos. Images of live embryos were acquired with a 20× immersion objective with glycerol oil (NA 0.7, *z*-step 1.19 µm). The 20× dry objective (NA 0.75, *z*-step 0.79 µm) was used for all fixed samples except for her9-MO experiments, which were acquired with a 20× immersion objective with glycerol oil (NA 0.75). The image format was 1024×512 and the scan speed ranged from 400 to 600 Hz. The software zoom was between 0.85× and 1.65×.

### Single clonal color analysis

Constructs for *her9* loss- and gain-of-function (LOF/GOF) analyses contained the UAS sequence (H2BCitrine:UAS:her9DN/her9FL) to conditionally alter the function of Her9 specifically in hindbrain boundary cells by injecting them into the Tg[BCP:Gal4] line (Hevia et al., 2022). For LOF experiments, a dominant-negative form of Her9 was generated by removing the WRPW domain, necessary for the transcriptional repressor activity of Her9 (her9^ΔWRPW^, her9DN in the text; Fisher et al., 1996; McLarren et al., 2001). For GOF, we cloned the full-length *her9* gene (her9FL). her9FL and her9DN were amplified by PCR from *her9* cDNA plasmid (Leve et al., 2001) with Phusion polymerase (Thermo Fisher Scientific) using the primers in Table S1. PCR products and the H2BCitrine:UAS plasmid (Nikolaou et al., 2009) were digested with XhoI and NheI, and ligated with T4 DNA ligase (Takara Bio) in a 5:1 (insert:vector) ratio. H2BCitrine:UAS, H2BCitrine:UAS:her9FL or H2BCitrine:UAS:her9DN were microinjected into Tg[BCP:Gal4] embryos at the one-cell stage at 15 ng/µl with *Tol2* mRNA at 18 ng/µl in a 1nl drop. For the analysis, the number of cells expressing H2BCitrine in each boundary were annotated. In H2BCitrine:UAS:her9DN/her9FL conditions, we quantified the percentage of boundary cells expressing each gene of interest over the number of H2BCitrine-cells exhibiting overexpression or ectopic expression of *her9.*

### Multicolor clonal tracking and analysis

#### Design and cloning

For functional multicolor clonal analysis, we designed two zebrabow2.0 UAS constructs co-expressing either her9DN or her9FL with one of the fluorescent proteins of a quadrichromatic Brainbow cassette modified from that described by Loulier et al. (2014). The two zebrabow2.0 constructs (UAS:her9DN-zebrabow2.0 and UAS:her9FL-zebrabow2.0) express by default the far-red protein iRF670 (Shcherbakova and Verkhusha, 2013) fused to the zebrafish histone gene *h2az2a* (Wilkinson and Shyu, 2001) to direct expression in the nucleus; upon recombination, expression switches to one of the three spectrally distinct fluorescent proteins: mEYFP (Zacharias et al., 2002), mTurquoise2 (Goedhart et al., 2012), or tdTomato (Shaner et al., 2004). her9DN and her9FL were separated from the tdTomato by the zebrafish cleavage peptide P2A (Kim et al., 2011). These transgenes enable equilibrated expression of the three recombination outcomes independently of Cre dosage and allow the comparison of the behavior of her9DN- or her9FL-expressing clones (tdTomato positive) with wild-type clones (tdTomato negative) within the same embryo. The designed sequence was synthesized in a pUC57-mini vector (GenScript Biotech). This plasmid was also designed as a versatile functional tool to allow the insertion of any gene of interest and the modification of the different fluorescent proteins by restriction enzymes. For the *her9* gain of function (UAS:her9FL-zebrabow2.0), her9FL was amplified as described above using primers listed in Table S1, and cloned within BshTI and XmaJ sites.

#### Injection

Tg[BCP:Gal4] embryos at the one-cell stage were injected with UAS:zebrabow1.0 (Pan et al., 2013), UAS:her9DN-zebrabow2.0 or UAS:her9FL-zebrabow2.0 (see before) at 10 ng/µl with *Tol2* mRNA at 18 ng/µl and 0.25 µl Cre recombinase at 15,000 units/ml (New England Biolabs, M0298M) in 4 nl drop as described by Brockway et al. (2019). For multicolor functional experiments, we injected 0.5 µl of Cre to increase recombination efficiency.

#### Imaging

Embryos were selected and imaged *in vivo*. For the wild-type clonal study, live embryos were imaged every hour from 32 (t0) to 48 (tf) hpf at 23°C in independent imaging sessions. The embryonic stage was corrected according to the temperature used for imaging. For the functional study, embryos were *in vivo* imaged at 36 and 48 hpf. Between imaging sessions, embryos were demounted and grown at 23°C. In these experiments, we also acquired the brightfield channel to observe the contour and landmarks of the hindbrain.

#### Image processing

Images were rotated to align the hindbrain midline with the *x*-axis. In timelapses, a drift correction was performed using a modified version of the script used by Dyballa et al. (2017). A region of interest (ROI) was used to include the whole hindbrain boundary in all time frames (timelapses) or individual clones at fixed points (functional analysis). Images were then converted to .xml/.hdf5 using the Big Data Viewer Plugin.

#### Color analysis

To establish the clonal criteria in the multicolor time-lapse data, we analyzed color frequency to identify clones with less frequent colors to define the characteristics for all tracked clones. Cell centers within boundary ROIs were pointed and saved using ROI Manager in ImageJ. An average of red, green, and blue channel values was determined by semi-manual ROI measurements and a ternary plot was generated using ImageJ macros adapted from Loulier et al. (2014). In all embryos, the number of cells in each of the 25 color identities defined according to the subdivisions of the RGB space was quantified (Fig. S4A). Color identities higher than 10% were considered frequent, whereas colors lower than 2.5% were considered rare (Fig. S4B,C). Based on the size and cell fate of rare color clones, we followed boundary clones located in the progenitor domain at t0 (32 hpf) that contained one to three cells per clone. Color analysis was further used to confirm the clonal relationship of cells. Cells falling in the same RGB subdivision and in close contact were considered clonally related (Fig. S4A,B). Boundary clones with frequent colors were only followed when isolated. To represent the applied color and the followed spatial criteria, we generated a 3D-ternary plot in the mediolateral (ML) axis using a MATLAB code (Fig. S4A, right panel; Loulier et al., 2014). For the functional study, we followed the same color analysis to establish the clonal relationship, and the mean intensity of the red channel to classify her9DN/her9FL (red) and control (non-red) clones. Clones were considered red when displaying more than 5% of red intensity, and non-red when displaying less than 1% (Fig. S4E,F). Clones that did not fall into these categories were excluded from the analysis.

#### Cell counting and tracking

For the timelapses, cells were annotated and tracked in MaMut software v.0.30.0 (Wolff et al., 2018) from t0 (32 hpf) to tf (45 hpf). For these analyses, we tracked 44 clones in different hindbrain boundaries of four different embryos (r2/r3, r3/r4, r4/r5 and r6/r7 in e10_2205; r2/r3, r3/r4, and r4/r5 in e1_3005; r3/r4, r4/r5, and r5/r6 in e3_1505; and r4/r5 in e7_2503). A number was ascribed to each clone in a boundary according to its ML position (from left to right). All clones, cell lineages, and cell divisions were displayed as lineage trees obtained from the MaMut Track Scheme and posteriorly drawn in Illustrator (Adobe) (Fig. S4D). For the functional analyses, cells were annotated in MaMut software v.7.0.2 (Wolff et al., 2018). We analyzed a total of 12 embryos at 36 hpf (21 non-red versus 20 red clones) and 15 embryos at 48 hpf (18 non-red versus 19 red clones) for the *her9* loss-of-function condition, and 13 embryos at 36 hpf (17 non-red versus 19 red clones) and 11 embryos at 48 hpf (11 non-red versus seven red clones) for the *her9* gain-of-function. Clones were ascribed a number in each boundary according to their ML and dorsoventral (DV) position. The same number was used to link the data of the same clone from 36 hpf to 48 hpf.

#### Cell fate, proliferative capacity, and cell division modes analyses

For clonal analyses we annotated: (1) the number of cells at 32 hpf and 45 hpf; (2) the number and time of cell divisions per clone in the wild-type tracked clones; (3) the cell fate at 45 hpf [progenitor (P) versus neuron (N)] by the relative position to the ventricle based on the method of Hevia et al. (2022) and immunostainings of neural progenitor (Sox2) and neuronal (HuC; Elavl3) markers performed at 45 hpf (Fig. S4A), and by the presence of the apical contact (a cell was considered a progenitor when the nucleus was close to the ventricle and showed an apical contact, or a neuron if the nucleus was close to the mantle zone and had no apical contact; Fig. S4A, spatial criteria); and (4) the cell division mode (PP, PN, NN). The cell division mode was determined according to the defined fate and relative position of daughter cells. A division was considered PN when the two daughter cells were not in close contact, with one closer to the ventricle and the other to the mantle zone; and a division was considered PP or NN when both daughter cells were in close contact and located at the same DV level. For the cell division mode analysis across time, fate was not ascribed for cells that were tracked less than 3 h after division. In the functional analysis, the same criterion was followed. The number of PP divisions in a clone was defined by the number of progenitors in the clone, as every PP division provides one new progenitor. The number of PN in a clone was established by the number of neurons in the clone, as every PN division provides one new neuron. Only clones composed of an even number of neurons at 36 hpf were considered to undergo NN divisions.

### Quantification and statistical analyses

#### Cell counting

We defined different independent experimental units according to the experimental set ups: (1) embryos, in the cases of MO or sgRNA injections_,_ and cell proliferation experiments in which we analyzed the effects at the whole cell population level; (2) hindbrain boundaries, in the single-color analysis since we could not segregate single clones; and (3) clones, in the multicolor functional analysis, in which we examined single and isolated clones. In the MO and sgRNA experiments, we counted the number of boundary cells (labeled by the Tg[BCP:Gal4]) in the r4/r5 boundary, and for single-color and multicolor clonal analyses we quantified boundary cells in the r2/r3, r3/r4, r4/r5, and r5/r6 boundaries. Cells were counted within an ROI including the whole boundary. The ROI was defined for each experiment and the same one was used for the control and the experimental group. For the proliferation analysis at late stages, the established ROI included the whole hindbrain. The quantification was performed in all the *xy* planes contained within the ROI (Fig. S2M). We counted the cells using the MaMut Fiji Plugin v.7.0.2 (Wolff et al., 2018) and annotated the number of boundary cells expressing the gene or marker of interest.

#### Statistical analyses

We performed normality tests and the corresponding *t*-test (Welch's test) or non-parametric test (Mann–Whitney test) according to the distribution of the data. To compare more than two experimental groups, we performed one-way ANOVA with Dunnett's multiple comparison test. Values are expressed as mean±s.d. All graphs were generated with GraphPad Prism 8 software. Image brightness and contrast were linearly adjusted in ImageJ. Median filters were applied in images in Fig. 1 and Fig. S1 only for presentation purposes. The Bleach Correction Plugin of ImageJ was used for the red channel in the multicolor time-lapse example (Movie 1) for better representation. All figures were assembled in Photoshop (Adobe v.23.5) and illustrations were generated in Illustrator (Adobe v.27.3).

## Supplementary Material



10.1242/develop.203164_sup1 Supplementary information
