# Supplementary material for: Her9 controls the stemness properties of hindbrain boundary cells
Source: Development. 2025 Jan 2;152(1):dev203164. doi: 10.1242/dev.203164 (PMC11829766; doi:10.1242/dev.203164)
Supplement: Supplementary information [file develop-152-203164-s1.pdf]

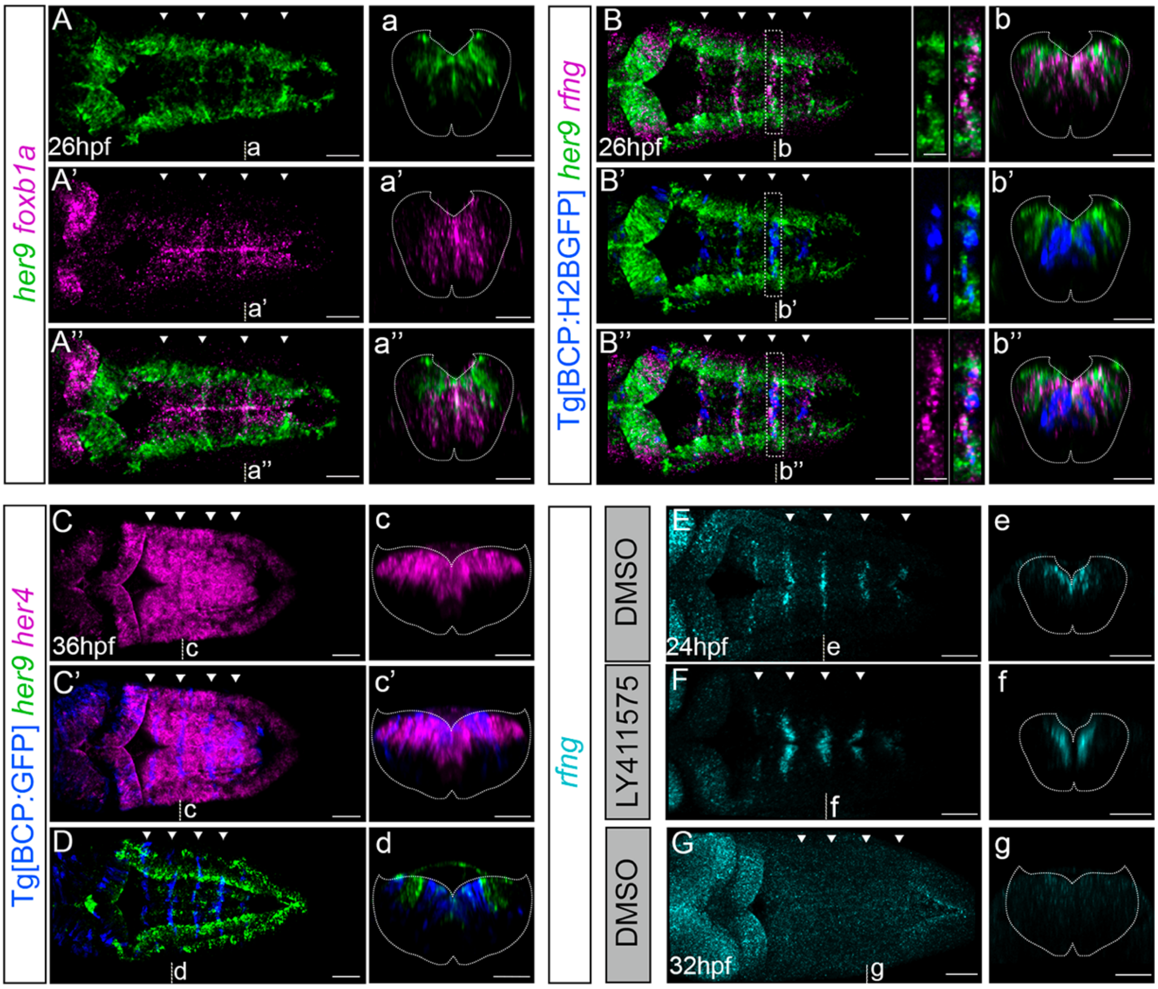

Fig. S1. Enriched *her9* expression overlaps with hindbrain boundary markers.
(A–A'', B–B'') *In situ* hybridization of *her9* and the *foxb1a* (A'-A'') or *rfng* (B, B'') boundary markers in Tg[BCP:GFP] or Tg[BCP:H2BGFP] (B'-B'') embryos at 26hpf. Dorsal Maximal Intensity Projections (MIPs) of single (A–A') or merged channels (A'', B–B''). Magnifications of one boundary displaying a single or merged channels. (a–a'', b–b'') Transverse projections of (A–A'', B–B'') through r4/r5 boundary. (C–D, C') *In situ* hybridization of *her4* or *her9* in Tg[BCP:GFP] embryos at 36hpf. Dorsal MIP of single (C) or merged channels are shown (C', D). (c–d, c') Transverse projections of (C–D, C') through r3/r4 boundary. (E–F) Embryos treated from 18hpf to 24hpf either with DMSO (E) or with LY411575 (F) and *in situ* hybridized with *rfng*. (e–f) Transverse projection through r4/r5. (G) *In situ* hybridization of *rfng* in Tg[elA:GFP] embryos at 32hpf. (g) Transverse projection (G) through r4/r5. Arrowheads indicate the position of the hindbrain boundaries. Dotted line delimitates the contour of the neural tube. BCP, Boundary Cell Population; hpf, hours post fertilization. Scale bar 50µm.

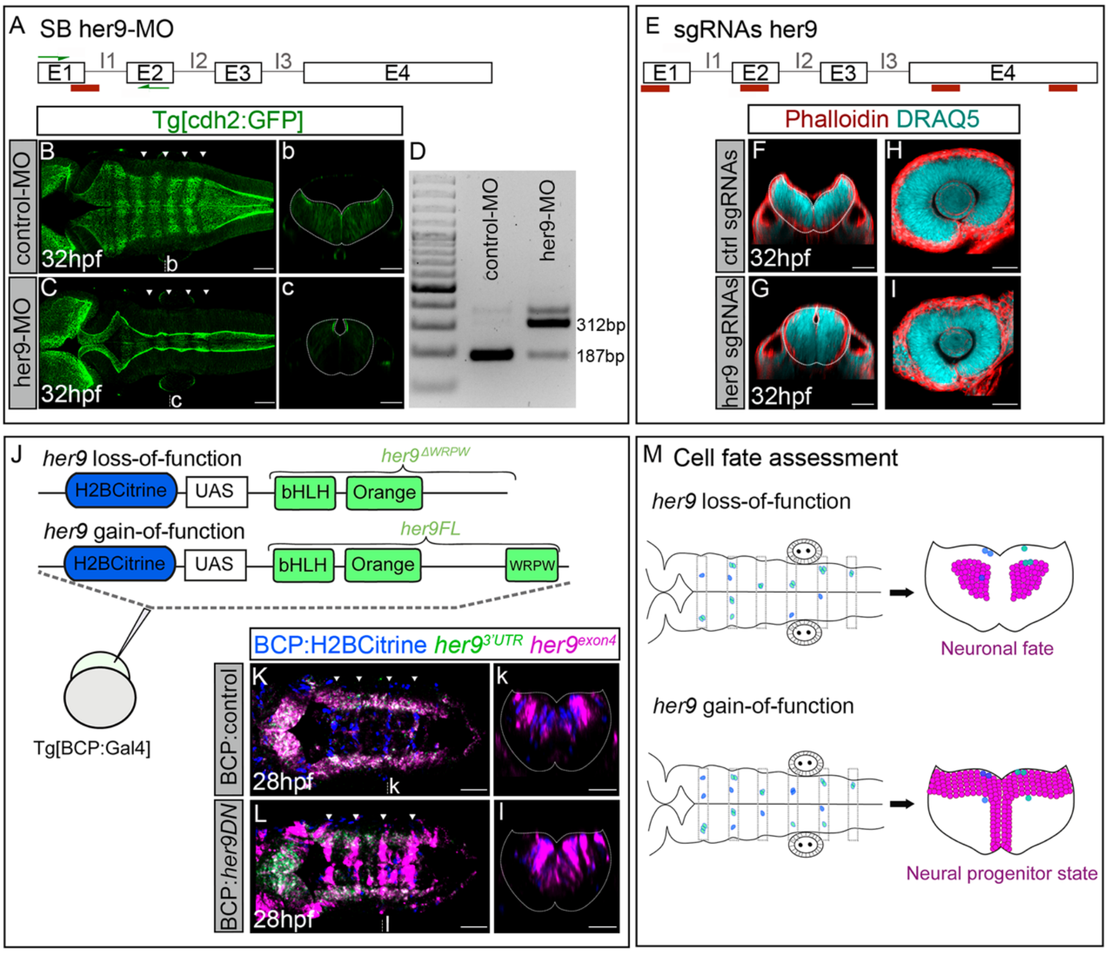

Fig. S2. Loss-and gain-of-function of *her9*.

(A) Scheme depicting the structure of the splicing blocking *her9* morpholino, SB her9-MO. Position of the her9-MO (red box), and the primers to assess its efficiency (green arrows). E1–E4, exon 1–4; I1–I3, intron 1–3. (B–C, b–c) Tg[cdh2:GFP] embryos injected with control-MO or her9-MO at one cell stage. Note that Her9 downregulation resulted in defects in the opening of the hindbrain ventricle (n=10/13), whereas no defects were observed in controls (n=8/8) at 32hpf. (B–C) Dorsal views with anterior to the left, (b–c) transverse projections of (B–C) through r4/r5 boundary. Arrowheads indicate the position of the hindbrain boundaries. Dashed line delimitates the contour of the neural tube. Scale bar 50µm. (D) Agarose gel showing the *her9* spliced-defective variant detected by RT-PCR in control-MO or her9-MO embryos in (B–C). (E) Scheme depicting the position of the four different sgRNAs used to redundantly target *her9* (red boxes). (F–I) Embryos injected with control or *her9* sgRNAs at one cell stage and stained with the nuclear marker DRAQ5 and plasma membrane marker Phalloidin at 32hpf. Note that Her9 depletion resulted in defects in the opening of the hindbrain ventricle (n=5/5) and smaller eyes (n=8/10), whereas no defects were observed in controls (n=5/5 and n=9/9, respectively). (F–G) Transverse projections of r4/r5. (H-I) Sagittal projections of the eye. (J) Scheme depicting the constructs and the strategy for conditional

loss- and gain-of-function experiments. Tg[BCP:Gal4] embryos at one cell stage were injected with either H2Bcitrine:UAS:her9DN (LOF, $her9^{\Delta WRPW}$) or H2Bcitrine:UAS:her9FL (GOF) to specifically express them in boundary cells. (K–L) Tg[BCP:Gal4] embryos injected with H2Bcitrine:UAS (K) or H2Bcitrine:UAS:her9DN (L), and *in situ* hybridized with *her9* 3'UTR (green) and *her9* exon4 (magenta) to distinguish endogenous (green and magenta) from exogenous (only magenta) expression at 28hpf. (K–L) Dorsal MIP of the hindbrain with anterior to the left, with transverse views of r4/r5 (k, l). Scale bar 50µm. (M) Cell fate assessment according to co-expression with neuronal fate and neural progenitor genes. To determine boundary cell fate in *her9* LOF or GOF, ROIs containing the whole stack (dotted gray frames) of each hindbrain boundary were analyzed.

Development • Supplementary information

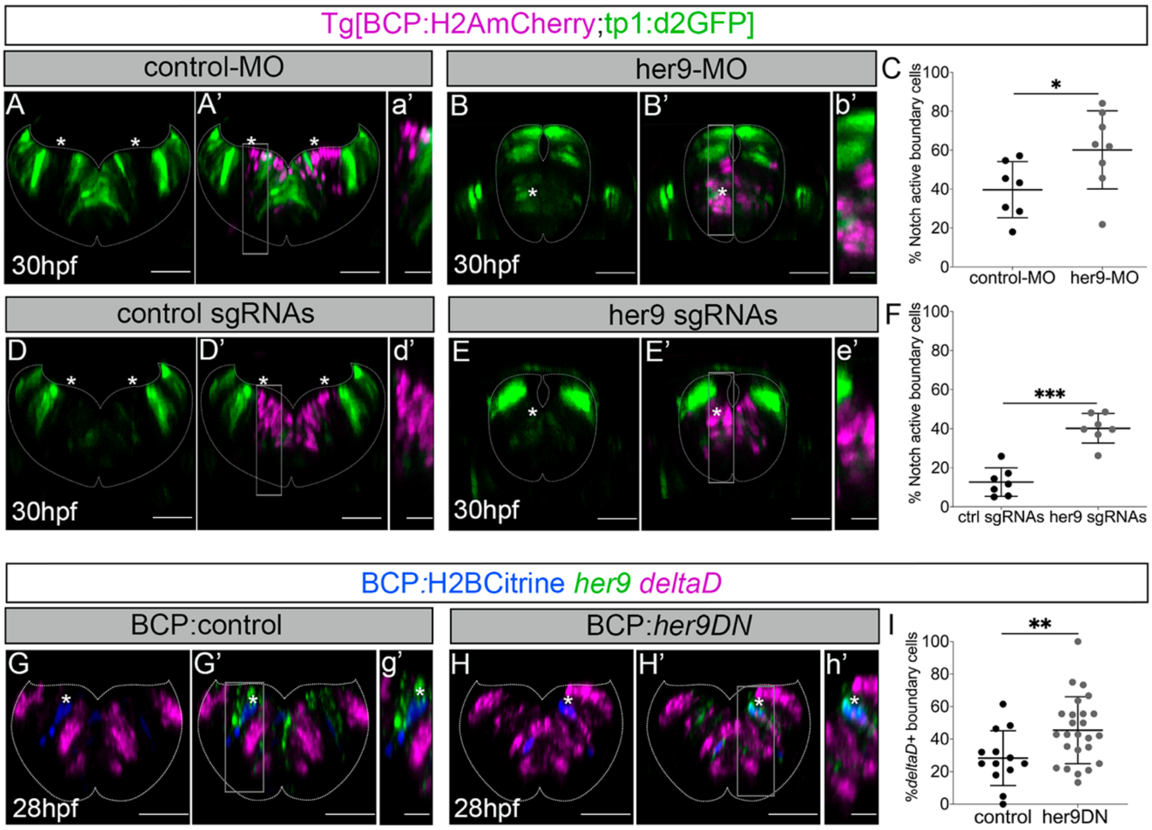

Fig. S3. Her9 prevents the onset of Notch activity at early embryonic stages.
(A–F, A'–B', D'–E') Tg[BCP:H2AmCherry;tp1:d2GFP] embryos injected either with control-MO (A) or her9-MO (B), or with control sgRNAs (D) or *her9* sgRNAs (E), displaying Notch activity and the boundary cell nuclei at 30hpf. Transverse projection of r4/r5. (a'–b') Magnifications of the gray framed regions in (A'–B', D'–E'). (C) Plot showing the Notch active boundary cells in control-MO and her9-MO injected embryos (39.7%±14.4 in control-MO n=7 vs. 60.1%±20.1 in her9-MO n=8; p=0.04[*], Welch's test). (F) Plot showing the Notch active boundary cells in control sgRNAs or *her9* sgRNAs injected embryos (12.7%±7.3 in control sgRNAs n=7 vs. 40.2%±7.6 in *her9* sgRNAs n=7; p<0.0001[***], Welch's test). (G–I, G'–H') Tg[BCP:Gal4] embryos injected with H2Bcitrine:UAS or H2Bcitrine:UAS:her9DN and *in situ* hybridized with *her9* and *deltaD*. (G–H, G'–H') Transverse single-sections of r3/r4 or r4/r5, respectively. (g'–h') Magnifications of the gray framed regions in (G'–H'). (I) Plot displaying the percentage of boundary cells expressing *deltaD* [28.3%±16.9 (of 29±17.5 cells) in controls, n=13 boundaries, N=6 embryos vs. 45.5%±20.6 (of 19±11.6 cells) in her9DN embryos, n=25 boundaries, N=10 embryos; p=0.009[**], Welch's test]. The plots show the mean±SD. Dashed line delimitates the contour of the neural tube. BCP, Boundary Cell Population; hpf, hours post fertilization; MO, morpholino. Scale bar 50μm and 20μm for magnifications.

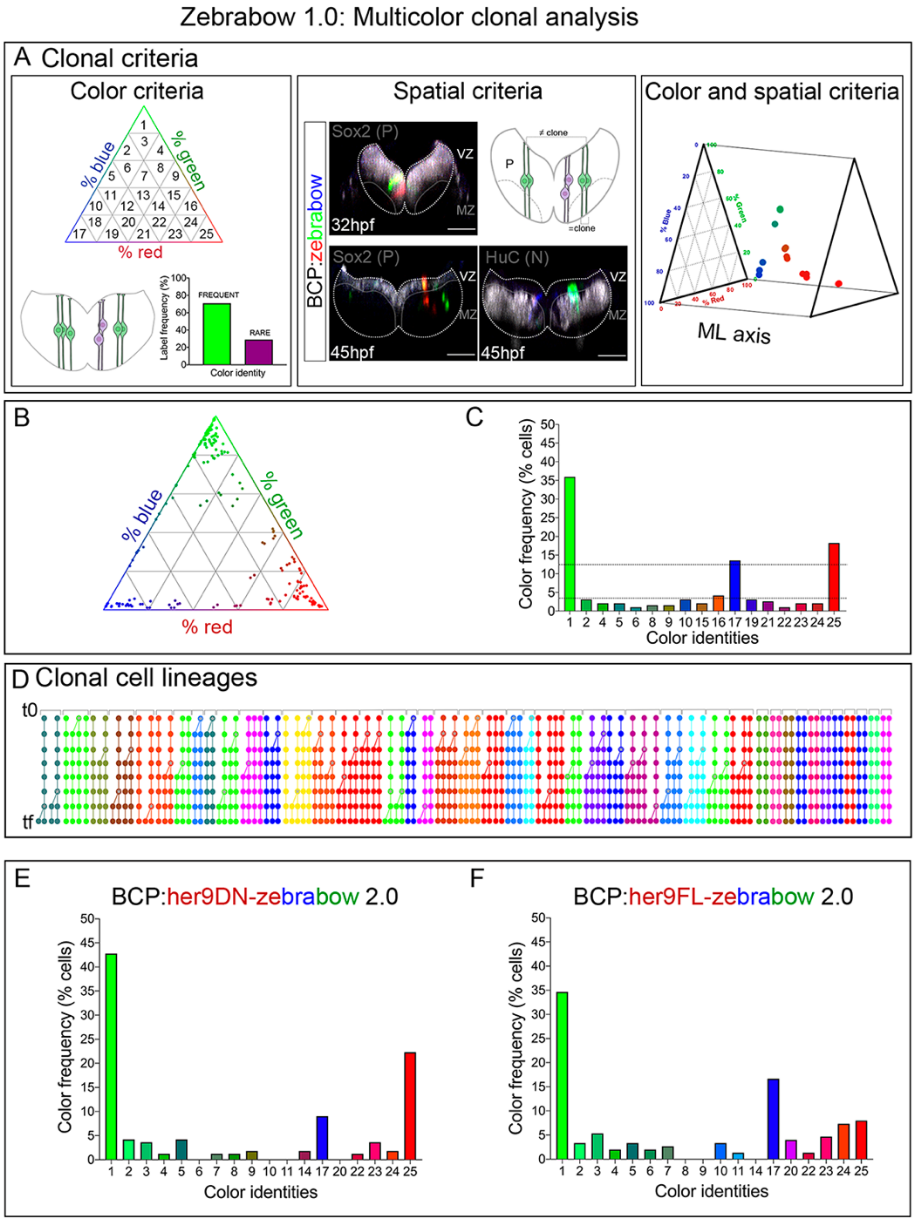

Fig. S4. Multicolor clonal tracking using the zebrabow1.0/2.0 systems.

(A) Criteria based on cell color and position used for clonal identification. For the color criterion (left panel), we analyzed the frequencies of color label combinations expressed by boundary cells and used the rarest colors to establish the size and fate criteria at 32hpf (t0). The histogram plot shows an illustrative example of clones displaying frequent and rare colors. The spatial criterion (middle panel) considered whether cells were in the progenitor domain (located in the ventricular zone depicted by Sox2 expression) at 32hpf and whether cells with the same color were in close contact. The fate of boundary derivatives at 45hpf was determined for their position (either in the progenitor or in the neuronal differentiation domain depicted by Sox2 or HuC expression, respectively), the presence of the apical contact and relative position between cells in the clone. Both color and spatial criteria were used for the clonal assessment (right panel): 3D ternary plot including the mediolateral (ML) axis of

Development • Supplementary information

r4/r5 boundary at 32hpf corresponding to (Figure 3b). (B) Ternary plot showing the normalized RGB values (% of each fluorescent protein to the global signal) of 169 labeled cells from 4 different embryos (e1–e4) at t0 (32hpf). (C) Histogram displaying the frequency of the 25 color subdivisions defined in (A). The lower black dashed line indicates the 2.5% threshold under which colors were defined as rare. The upper black dashed line indicates the threshold of 10% above which labels were considered frequent. (D) Lineage trees of all the boundary cell clones tracked from 32hpf (t0) to 45hpf (tf) (n=44 clones, N=4 embryos; 90 cells at t0 and 138 cells at tf). Dots represent cells, lines connect the cells from the same track, and branch points indicate cell divisions. Cell lineages are color-coded according to the clone of origin. Time is represented vertically, with t0 (32hpf) at the top and tf (45hpf) at the bottom. (E–F) Histograms displaying the frequencies of color labels observed at 36hpf in BCP:her9DN-zebrabow2.0 and BCP:her9FL-zebrabow2.0 embryos, respectively. Y-axes represent the percentage of cells found in each of the 25 color subdivisions of the normalized RGB space (only expressed color subdivisions were plotted).

Development • Supplementary information

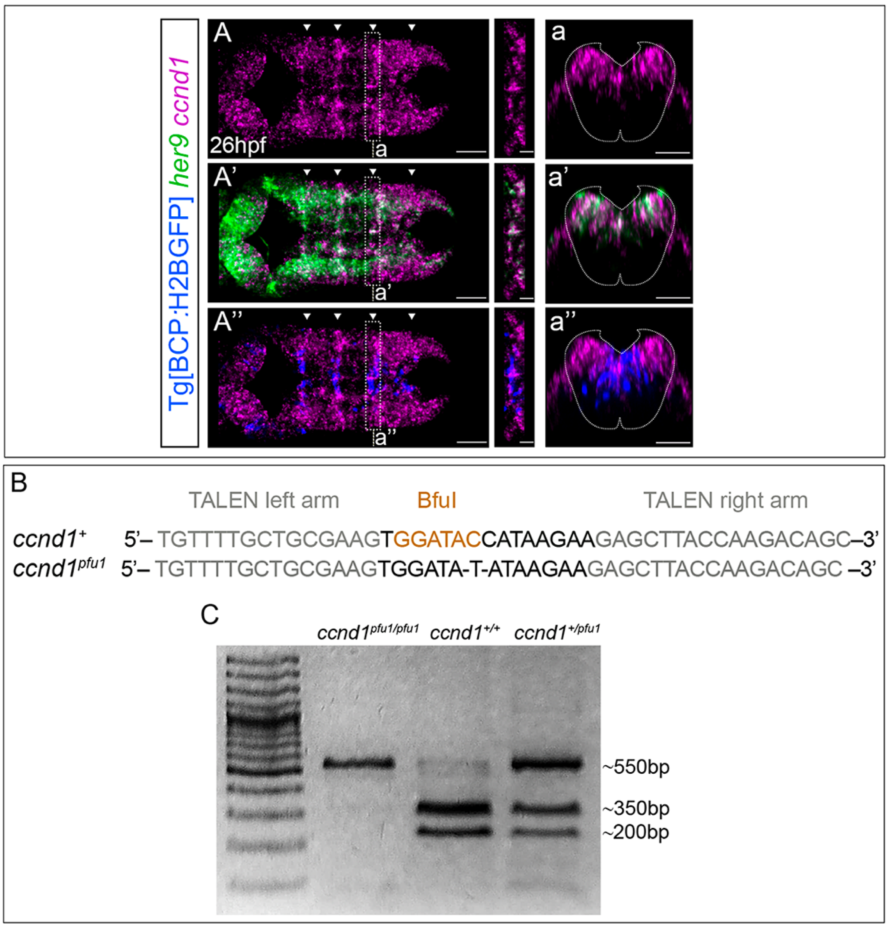

Fig. S5. Genomic edition of *ccnd1* gene by TALEN technology.

(A–A'') Dorsal MIPs of Tg[BCP:H2BGFP] embryo *in situ* hybridized with *ccnd1* and *her9*. Magnifications correspond to the dotted framed regions of the indicated boundary displaying single or merged channels. (a–a'') Transverse projections of (A–A'') through r4/r5 boundary. Arrowheads indicate the position of the hindbrain boundaries. Dotted line delimitates the contour of the neural tube. BCP, Boundary Cell Population; hpf, hours post fertilization. Scale bar 50µm. (B) Alignment of the *ccnd1^{pfu1}* mutant allele sequence with the wild-type allele (*ccnd1^{+}*) showing the deleted nucleotides as – and the TALEN target sites in the *ccnd1* locus. The left and right arms (grey) of these sites are separated by a spacer (black) including the restriction site used for screening (orange). (C) Agarose gel with the bands resulting from enzyme restriction analysis of embryos carrying the wild-type (*ccnd1^{+}*) or the mutated allele (*ccnd1^{pfu1}*). The size of the different obtained fragments is indicated. The *ccnd1^{pfu1}* allele generates a truncated cyclinD1 protein of 12AA.

Table S1. DNA sequences used for different experimental strategies.
Name and DNA sequences employed for the different used experimental strategies.

EXPERIMENT TYPE	NAME	SEQUENCE
**mRNA probe synthesis**	T3- *foxb1a* Fw	5' –GGC CCC ATA TGT ACA GCA CT– 3'
	T7- *foxb1a* Rv	5' –CAG TGC TTG GAT TTG TCG AA– 3'
	Sp6- *her4.2* Fw	5' –GCT CAA TCA GCA GCA GAG AA– 3'
	T7- *her4.2* Rv	5'–CAA ACC CAA TAT GGG TGA AA– 3'
	T3- *cdkn1ca* Fw	5' –AAC GTG GAC GTA TCA AGC AA– 3'
	T7- *cdkn1ca* Rv	5' –GTG CTG TTT CTG GGG CTC T– 3'
	T3- *ccnd1* Fw	5' –GCT CGA GGT CTG TGA AGA GC– 3'
	T7- *ccnd1* Rv	5' –GAC TTG CGA GAG GAA GTT GG– 3'
	T3- *her9* 3'UTR Fw	5' –TTG CGC ATC TCA TAT GAA CTT A– 3'
	T7- *her9* 3'UTR Rv	5' –TTT CGT TAT TTT GAT TTA TTC AGC A– 3'
	T3- *her9* exon4 Fw	5' –GAC ACA AAC GTC CTC AGC AA– 3'
	T7- *her9* exon4 Rv	5' –ACT GAC ACC AAC GGG ACT G– 3'
**H2Bcitrine:UAS:her9FL/ her9DN**	XhoI-*her9* Fw	5'–CCG CTC GAG ATG CCA GCC GAT AAT ATG GA– 3'
	NheI-*her9* Rv	5'–CTA GCT AGC CTA CCA GGG TCT CCA CAC CG– 3'
	NheI-*her9*$^{\Delta WRPW}$ Rv	5'–CTA GCT AGC CAC CGG CTC ATT GCT TTC TG– 3'
**UAS:her9FL:zebrabow2.0**	AgeI-Kozak-*her9* Fw	5'–TCG ACC GGT GCA AAC ATG CCA GCC GAT AAT ATG GA– 3'
	AvrII-*her9* Rv	5' –AAA CCT AGG CCA GGG TCT CCA CAC CGG CTC ATT GC– 3'
**her9-MO efficiency assessment**	MO-2 *her9* e1 Fw	5' –CCG GAC TCA ACT TTG GTG TT– 3'
	MO-2 *her9* e2 Rv	5' –AAG GCT CTC GTT GAT TCT CG– 3'
***her9* sgRNAs**	control sgRNA #1	5' –GGC TCC TTG CTC GCG ACT TG– 3'
	control sgRNA #2	5' –GGC GAC CGT GGC CGG AAC GA– 3'
	control sgRNA #3	5' –GCC TGC GCG TAC CGA CTC GG– 3'
	control sgRNA #4	5' –ACT CGA TTG TAC CTA GTG GT– 3'
	her9 sgRNA #1	5' –GAT CAT GCC AGC CGA TAA TA– 3'
	her9 sgRNA #2	5' –CGA GAA TCA ACG AGA GCC TT– 3'
	her9 sgRNA #3	5' –CGA TTT CTC TCT ACC TGC GA– 3'
	her9 sgRNA #4	5' –ACG GTC GCA TCT CCT GTC CA– 3'
***ccnd1*^{pfu1} genotyping**	*ccnd1* TALEN Fw	5' –GGG AGT TTT GTC AAG CGG AG– 3'
	ccnd1 TALEN Rv	5' –GGG ATG GAT AAG CAA TGC CG– 3'

Development • Supplementary information

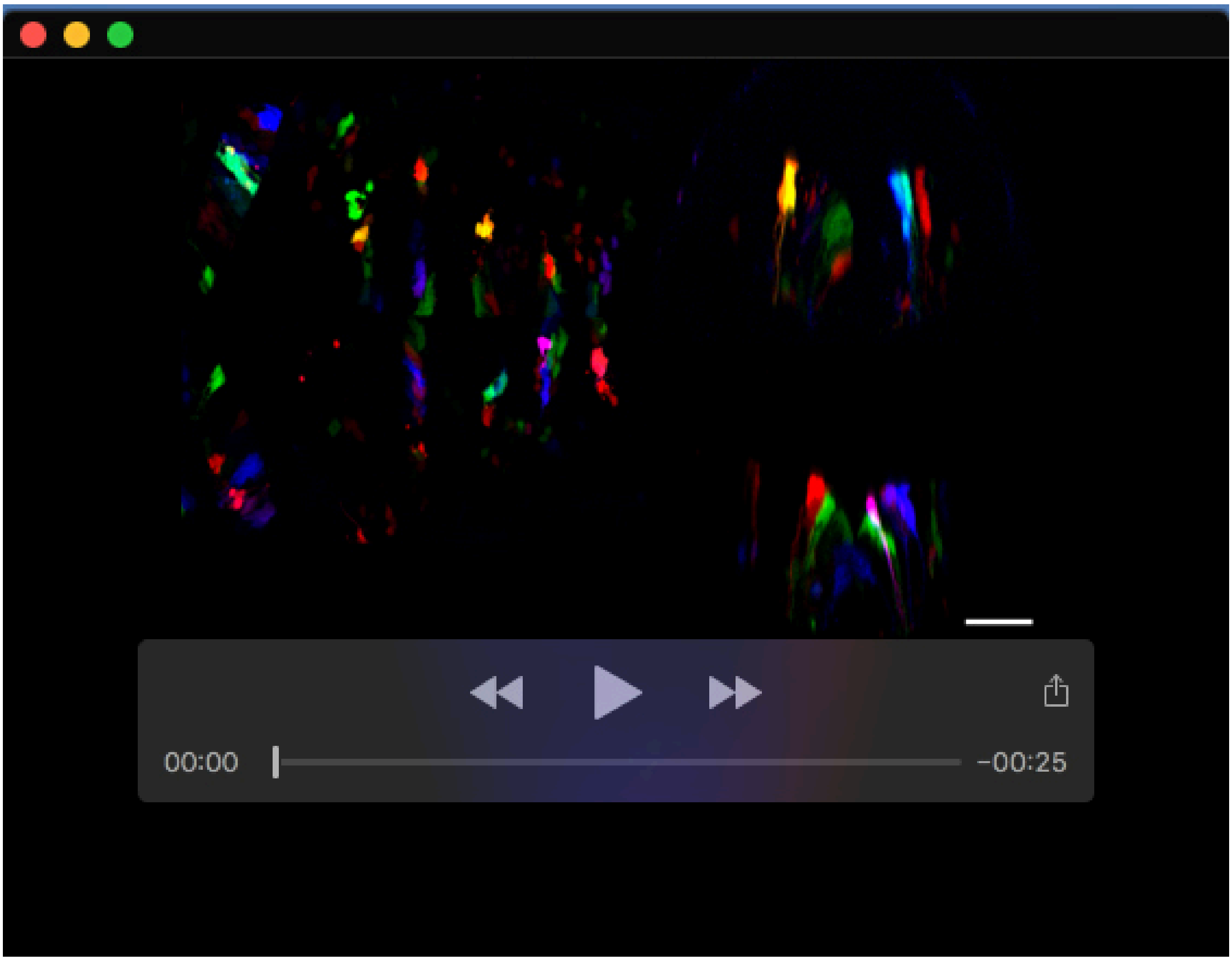

Movie 1. Time-lapse of boundary cells labeled with the zebrabow1.0 approach.
The video displays a hindbrain of a Tg[BCP:Gal4] embryo injected with Cre protein and the UAS:zebrabow1.0 construct containing the dTomato, mCerulean, and eYPF genes, imaged from 32 to 45hpf. (Left) Dorsal MIP and (right) transverse projection through r4/r5 (top) and r5/r6 (bottom). BCP, Boundary Cell Population; hpf, hours post fertilization. Scale bar 50µm.
