# Supplementary material for: Her9 controls the stemness properties of hindbrain boundary cells
Source: Development. 2025 Jan 2;152(1):dev203164. doi: 10.1242/dev.203164 (PMC11829766; doi:10.1242/dev.203164)
Supplement: Supplementary file 1 [file develop-152-203164_review_history.pdf]

Her9 controls the stemness properties of hindbrain boundary cells

Carolyn Engel-Pizcueta, Covadonga F Hevia, Adrià Voltes, Jean Livet and Cristina Pujades
DOI: 10.1242/dev.203164

Editor: Francois Guillemot

Review timeline

Original submission:	18 June 2024
Editorial decision:	22 July 2024
First revision received:	22 October 2024
Editorial decision:	12 November 2024
Second revision received:	16 November 2024
Accepted:	26 November 2024

Original submission

First decision letter

MS ID#: DEVELOP/2024/203164

MS TITLE: Her9 controls the stemness properties of hindbrain boundary cells

AUTHORS: Carolyn Engel-Pizcueta, Covadonga F Hevia, Adria Voltes, Jean Livet, and Cristina Pujades

I have now received the reports of three referees on your manuscript and I have reached a decision. The reports are appended below and you can access them online: please go to BenchPress and click on the 'Manuscripts with Decisions' queue in the Author Area.

As you will see, all the referees express great interest in your work, but they also have significant criticisms and recommend a substantial revision of your manuscript before we can consider publication. In particular, they ask you to address the following concerns: the lack of validation of the phenotypes obtained using morpholinos; the subtle and variable effects of Her9 manipulations; the quantification and interpretation of data in Fig. 2; the definition of progenitor cell fate using cell position in live imaging experiments; and the superficial analysis of the relationship between Her9 and Ccnd1. If you are able to revise the manuscript along the lines suggested by the referees, which may involve further experiments, I will be happy to receive a revised version of the manuscript. Your revised paper will be re-reviewed by the original referees and its acceptance will depend on your addressing satisfactorily all their major concerns. Please also note that Development will normally permit only one round of major revision.

If it would be helpful, you are welcome to contact us to discuss your revision in greater detail. Please send us a point-by-point response indicating your plans for addressing the referees' comments, and we will look over this and provide further guidance.

Please attend to all of the reviewers' comments and ensure that you clearly highlight all changes made in the revised manuscript. Please avoid using 'Tracked changes' in Word files as these are lost in PDF conversion. I should be grateful if you would also provide a point-by-point response detailing how you have dealt with the points raised by the reviewers in the 'Response to Reviewers' box. If you do not agree with any of their criticisms or suggestions please explain clearly why this is so.

© 2025. Published by The Company of Biologists under the terms of the Creative Commons Attribution License (https://creativecommons.org/licenses/by/4.0/).

Reviewer 1

Advance summary and potential significance to field

in zebrafish. Initial comparisons with her4 and assessment of the impact of blocking N activity show the her9 is initially N-independent and acts to maintain boundary cells in a progenitor state. her9 eventually downregulates at boundaries, coinciding with neuronal differentiation. The Zebrabow system is adapted to conduct lineage studies showing that there is no strong effect of clonal lineage on division patterns of daughter cells, nor is there a strong temporal signature to the incidence of symmetric P-P vs. asymmetric P-N divisions (although symmetric N-N divisions become more common at later stages). Boundary-specific misexpression of her9DN promotes neural differentiation whereas misexpression of her9FL promotes retention of progenitors. The authors then focus on ccnd1 at boundaries and cdkn1ca in flanking cells and find that her9 is required to block expression of cdkn1ca at boundaries, allowing cells to proliferate, whereas a ccnd1 knockout reduces boundary cells in a manner similar to her9-MO. Finally her9 marks radial glia at late stages (72 hpf) and is N-independent.

This is an interesting and potentially important study, with some notable strengths. It provides an elegant supplement to their earlier paper on progenitors in rhombomere centers and more generally reveals a very interesting transition in regulation of progenitors.

Comments for the author

Comments and recommendations:
1) Loss of function studies employed her9 morpholino instead of a mutant. This is disappointing given that her9 knockout alleles already exist. Validation of the MO is limited to confirming efficacy, but there is no assay to address specificity and potential off target effects. An alternative technique is regional misexpression of her9DN, but different assays are used for that construct. To help address the issue of specificity, it would help to show that her9-MO and her9DN produce similar phenotypes with the same assays. For example, in Fig. 2 check HuC expression after misexpressing her9DN, and check neurod4 and neurog1 expression in her9 morphants. Additionally, check the total number of BCP+ boundary cells in her9-MO, her9DN and her9FL.

2) In Fig. 2, it is shown that her9-MO increases boundary neurons at 30 hpf but not at 36 hpf. However the number of HuC+ neurons appears to be increased in Fig. 2E and F, despite statistical analysis suggesting otherwise. The control data in Fig. 2F appear strangely scattered, which of course impacts significance. How close to significance was the p-value? One wonders whether additional samples might achieve significance.

3) Expression of deltaD in Fig. S3 appears more widespread in controls (D, D') than in her9DN (E, E'). Please comment.

4) Figures 3 and 4 are very strong and clearly presented, but a few points of clarification would help. In Fig. 3 and relevant parts of the text, the beginning and ending time points are termed t0 and tf. This seems unnecessarily vague. It would be preferable to use unambiguous terms 32 hpf and 45 hpf respectively. In Fig. 4, her9FL appears to reduce cells per clone, yet it is concluded that her9 is "not sufficient for clonal growth". This may be strictly true, but it misses the point - it does not capture the essence of the data suggesting that excess her9 appears to reduce proliferation.

5) EdU incorporation is used to assess proliferative capacity (activity) and is described as a marker for cells in S-phase. This needs more explanation. Only a short pulse (a short incorporation time) would support this interpretation, whereas a long pulse would identify all cells that were recently cycling. Please clarify in the text. Description of EdU incorporation in Materials and Methods seems to suggest that embryos were placed on ice during EdU incorporation, as opposed to processing embryos on ice after some period of incorporation. Please clarify.

6) It is argued the her9 represses cdkn1ca at boundaries. The sections in Fig. 5 do not appear show particularly dramatic expansion of cdkn1ca into the boundary. It would likely help to show a dorsal view of cdkn1ca in her9 morphants, not just a section. The suggestion that cdkn1ca is a "mediator"

© 2025. Published by The Company of Biologists under the terms of the Creative Commons Attribution License (https://creativecommons.org/licenses/by/4.0/).

of her9 makes no logical sense. Expression patterns of these genes are complementary, and the regulatory relationship between them is far from clear. At most one could argue that expression of cdkn1ca in boundary cells might facilitate a change in mitotic activity after her9 is gone. Similarly, the suggestion that repressing cdkn1ca expression at the boundary permits transcription of ccnd1 is not logical. cdkn1ca may block ccnd1 activity at the protein level, but not at the level of transcription.

7) ccnd1 mutants are generated for this paper but are used for only a single assay: they show reduced numbers of BCP+ boundary cells, "similar her9 morphants". This is very cursory analysis and does not offer strong support for any conclusion. To make a stronger link to her9, it would help to show that ccnd1-/- eliminates or alters expression of her9, or other boundary markers, or allows cdkn1ca to be expressed at boundaries. It would also be useful to examine the effects of her9-MO (or her9DN) in ccnd1 mutants. If they are in the same pathway, or if ccnd1 is a downstream effector of her9, then there should be no phenotypic enhancement. This could also help validate specificty of her9-MO

8) The last part of Fig. 6 shows that her9-MO eliminates expression of a Yap/Taz reporter at boundaries.
The conclusion that these pathways act together to "synergistically promote proliferation" is not supported or warranted. Such a conclusion would require considerable additional experimental analysis with compound mutants. It is also not terribly convincing given the considerable variability of the reporter even in control embryos. This part of the paper is uncharacteristically weak and could be cut without detracting from the stronger parts of the paper.

Reviewer 2

Advance summary and potential significance to field

This manuscript explores the mechanism by which neurogenesis is suppressed at rhombomere boundaries in the zebrafish hindbrain. The authors identify her9 as being transiently expressed at rhombomere boundaries in a Notch-independent manner, where it prevents neurogenesis and promotes proliferation in part by suppressing expression of the cell cycle arrest gene cdkn1ca. To demonstrate this, the authors use an impressive combination of boundary-specific markers, boundary-specific expression of dominant-negative and full-length forms of her9, and lineage tracing. Their imaging approach, which is to make high-resolution X-Z projections of confocal image stacks, allows them to resolve single cells and their lineages in rhombomere boundaries. Consequently, the data is overall of high quality and although the phenotypes are sometimes subtle, they are well quantified and largely compelling.
Some aspects of these findings are known. For example, it was known that her9 and cdkn1c are expressed at rhombomere boundaries and that her9 suppresses neurogenesis there. These authors themselves previously quantified asymmetric and symmetric cell divisions using less elegant tools but with the same conclusions.
Nevertheless, the current paper shows all of these functions for her9 at better resolution and with better quantitation, and adds a novel single-cell-resolution timelapse analysis of cell fate decisions in vivo that is exceptionally elegant. I recommend publication in Development following the revisions suggested below.

Comments for the author

1) The authors use morpholinos rather than genetic mutants to evaluate her9 function in rhombomere boundaries. While this weakness is shored up partly by the nice use of boundary-specific dominant-negative expression, it remains an outdated technology that is prone to non-specific effects even if p53 Mos are co-injected as was done here. Multiple her9 alleles have been made in the zebrafish including a nonsense allele sa43978, available from the European stock center. The authors should confirm their boundary phenotype in her9 mutants by in situ or antibody staining. This is particularly important given that they co-inject a p53 MO to "diminish putative morpholino artifacts" but which could also alter real phenotypes relating to cell proliferation, cell survival and clone sizes.

© 2025. Published by The Company of Biologists under the terms of the Creative Commons Attribution License (https://creativecommons.org/licenses/by/4.0/).

2) The authors state that in Tg(BCP:Gal4) embryos all cells from the boundary cell population express Gal4 but this is clearly not the case; in fact expression is quite sportadic (Fig. 2). Neither her9 nor the BCP transgenic lines seem to be expressed in all of the cells at the rhombomere boundary, and in some images they seem to be almost mutually exclusive. This makes the interpretation of the experiments that use the BCP:gal4 line to drive her9DN and her9FL expression challenging. For instance, Fig. 4G shows that her9DN and her9FL expression both cause smaller clones. Could her9DN be causing smaller clones in BCP:Gal4 cells that normally express her9, while her9FL overexpression causes a phenotype in BCP:Gal4 cells that normally don't express her9? It would be helpful for the interpretation of these experiments if, in Fig. 2 the authors quantify the location and degree of overlap between her9 expression and the BCP driver expression at the early stage.
3) The method used for quantification of co-expression (Fig. 2) needs to be better explained. The authors state in the methods that an ROI is used that is the same size in experimental and control images, however it appears that the position of the ROI is not standard. In some images one side of the ROI is along the midline
(e.g. Fig. 2A'), but in others it is shifted away from the midline (e.g. Fig. 2B') and in some images the ROI includes cells on both sides of the midline (e.g. Fig. 2H'). This seems arbitrary when different choice of section e.g. in Fig. 2M, N would result in the opposite interpretation. What determined where the ROI was positioned in each image? Is it even necessary to use an ROI rather than just counting all the cells in the projection? It would be preferable to just include the whole section.
4) The authors should explain early in the text how they define the "progenitor domain". This is important because later in the paper they use positional criteria to define cell fate, in the absence of any marker gene expression.

5) Fig. 5V,W: it is unsurprising that knocking out a cell cycle gene would reduce the number of cells of any type. The connection to her9 is not direct. Furthermore, a clear prediction of the model would be that ccnd1 and her9 would be co-expressed in boundary cells however this is not evident from Fig. 5T.

Minor comments:

1) In the methods section titled "zebrafish strains" please include the ZFIN genomic feature name of each published transgenic line (e.g. Tg[tp1:d2GFP] mw43Tg).

Reviewer 3

Advance summary and potential significance to field

See attached doc

Comments for the author

Summary of the advance made in this paper and its potential significance to the field.
The paper deals with a fundamental problem in developmental neurobiology- how the spatiotemporal distribution of progenitors and neurons is being governed in the neural tube. This paper targets a unique cell population in the zebrafish hindbrain, as it is composed of two distinct domains- the rhombomeres and the inter-rhombomeric boundaries, while focusing on the characteristics of the later as transient non-neurogenic progenitors. To decipher what maintains the hindbrain boundary cells in such a state, the authors focused on the role of Her9 (the zebrafish homolog of the mammalian Her1). Previously, the same group showed that the temporal shift in the state of the boundary cells relies on Notch activity, causing them to transition from neuroepithelial progenitors to radial glia, along with changes in cell division patterns. Eventually, these cells undergo neural differentiation, contributing to the overall neurogenic capacity of the hindbrain. The current paper nicely continues in the same vein, presenting the finding that Her9 does no dependent on Notch signaling, whereas it inhibits the onset of Notch initiation and, consequently, the neurogenic process. In their evaluation of Her9 activity, the authors also demonstrate its effect on cell proliferation, highlighting two relevant cell cycle regulatory genes. Additionally, they provide a characterization of the clonal cell- division behavior of the boundary

© 2025. Published by The Company of Biologists under the terms of the Creative Commons Attribution License (https://creativecommons.org/licenses/by/4.0/).

cell populations using an advanced multicolor clonal analysis system. While new information is being provided on the dynamic spatiotemporal activity of Her9 in sustaining the inter-rhombomeric boundary cells in a non-differentiated state, the level of innovation is somewhat moderate as a very similar role of Her1 has already being shown in mice (Baek et al., 2006). Moreover, concerns arise regarding the choices of stages and the demonstration of effects, which in some cases lead to conclusions not strongly supported by the data. Hence, in my view, substantial revisions are needed to make the study at the appropriate level for Development.

Major comments

1. This is a general remark for an issue that proceeds along the entire manuscript. Embryonic stages differ between different experiments, and the choices of stages is often not clear. For instance, the authors show that her9 is expressed at the boundaries from 18-30 hpf, and claim that it acts to prevent boundary cells from adopting a neurogenic character at early stages. Yet, in most figures, the readout of Her9 modulations is presented at later stages. One example is in fig. 2, where most effects of her-9 downregulation/OE are shown at 30-36 hpf. If the authors could demonstrate that, upon her9 LOF, a precocious shift of boundary cells into neurogenic fate is evident at stages earlier than the ones shown in the paper, and that this effect is coupled with change in cell division patterns, this will provide much stronger support to the conclusions that her9 is mandatory to maintain the stemness of the cells at <u>early stages</u> when it is largely expressed at rhombomere borders. Careful selection of the most-relevant stages for analysis is therefore needed throughout the manuscript in order to support the conclusion on the fundamental role of her9 at early stages of development.

2. In figures showing expression pattern of her9 in whole mounts (such as figs. 1,5,6), it is often difficult to see the expression that is described in the text. This raises questions, since for instance in fig.2, her9 expression seems punctuated at these sites, and not fully overlapping with the boundary marker rfng. In fig. 5, the authors claim that her9 overlaps with ccnd1, but again, these patterns are not clearly evident in the whole mounts. Can they provide better staining/ higher magnifications? Alternatively, can the punctuated her9 expression may suggest that it is present and active only in a sub-group of boundary cells? If this is the case, this may explain the various quantification data that demonstrate only moderate changes in cell number which adopts different fates upon her9 manipulation (see Point 4).
3. Furthermore, when showing changes in neural gene expression upon downregulation/GOF of her9, the data is largely presented as a single virtual section (as max intensity projection) where only few cells are shown (i.e., fig.2). Whole mount views that demonstrate the effects on the entire tissue are missing, which limits the ability to be fully convinced by the data, especially since the quantifications show only mild changes in the percentage of cells expressing each marker in the treated vs. control hindbrains. It is not clear why they chose not to present the whole mounted hindbrain in fig.2, while in other cases they do show effects both in overviews and in sections (i.e., figs.5,6).
4. In figs. 2,5- since MO in zebrafish leads to total knock down of the Her9 gene, it is puzzling that we see such slight increases in the percentage of boundary derived neurons (for instance, fig.2C,F). What happens to the rest of the Her9-deviod boundary cells? This notion also implies to the her9DN results. Also, can they explain why on the her9DN only a portion of the DN-expressing boundary cells shifted towards neural differentiation, whereas in the her9OE a much greater proportion of cells remained expressing the progenitor gene Sox2?
5. The data presented in figs.3,4 is under the theme "Her9 controls the behavior of boundary cells", based on the documentation of the symmetric/asymmetric division of individually-labeled boundary cells. However, it is difficult to tell if a clone is a progenitor or neuron based on the images provided and without other appropriate markers (for instance, HuC/Sox2). Also, if the analysis was done based on the cells' location along the ventricular-mantle axis, can they show how they located accordingly rather than just showing colored cells with no context? Also, does her9 manipulations lead to the expected changes in cell morphology (i.e., radial glia etc) ?
6. As already noted in point 1, following her9 manipulation (fig.2), expression of HuC, neurod4 and neurog1 are analyzed at stages 36 and 28, respectively. If they claim that her9 downregulation leads to premature neurogenesis in boundary cells, why not showing earlier stages to support that claim? Also, if there are shifts in the progenitor state of the boundary cells, they should include a progenitor marker, along with the neurogenic markers provided in the same embryos, as part of the analysis.
7. In fig. 5- it doesn't look like there are less boundary cells in the Her9-MO in the images

© 2025. Published by The Company of Biologists under the terms of the Creative Commons Attribution License (https://creativecommons.org/licenses/by/4.0/).

provided. Also in the same section, if CCND1 is affected by Her9 activity in the boundaries, why wasn't its expression assessed upon Her9 MOs or in the Her9 constructs? Moreover, the reduction in the proportion of Edu+ cells in the her9 morphant is quite small. As her9 is globally downregulated, what can be the reason for this small effect? Intriguingly, the effect of her9KD on cell cycle genes/Edu uptake in their study is in discrepancy to that effects found in the hes1KO mice.

8. In its present form, fig.6 contains expression pattern/Edu labeling in the rhombic lip, without any causal link to her9. Based on all the tools developed for the previous experiments, it is not clear why the examined parameters (Sox2/fabp7a expression, mitotic events) were not tested following loss of Her9, since Her9 is claimed to be highly expressed in those lateral domains at the more-advanced stages. Is it possible that since it become confined to these lateral domains at later stages, rather than the boundaries, its role in hindbrain changes to promote radial glia fates?

Minor

4. In some assays, quantification is not provided and should be added.

2. Regarding the her9 loss and gain of function assays, no complementary assays are provided to prove that their constructs work as expected (as done for the MO).

3. The possibility of off-target effect of the her9MO has not been ruled out (by co-injecting p53MO for instance).

First revision

Author response to reviewers' comments

We thank the Reviewers and the Senior Editor for commenting on our manuscript. We have followed their advice and implemented most of their suggestions. We modified and improved the previous figures and generated a new one to include all new data to better support our findings. We provide an amended manuscript that I wish it will be suitable for publication in Development.

Before geOng into the specifics for each of the Reviewers, let me provide some background. To back up our results with the morphants and other funcQonal approaches, iniQally we tried to import the available *her9*$^{-/-}$ CRISPR mutants (Coomer et al., 2020), which harbor 1bp mutaQon causing a frameshiV resulQng in a premature stop codon. They are similar to the *her9*^{sa43978} mutants generated by Sanger SL7 screen (1bp mutaQon) menQoned by Reviewer 2, with the advantage that the *her9*$^{-/-}$ CRISPR mutants are already characterized. We aimed to cross the *her9*$^{-/-}$ mutants with the fish transgenic line (Tg[BCP:H2AmCherry]; (Hevia et al., 2022) to have a landmark of the boundaries and therefore the morphological context. We failed to do this since no embryos survived upon shipping from the US as it oVen happens. Thus, we conQnued our strategy to combine disQnct funcQonal approaches such as generaQng her9 morphants and performing condiQonal funcQonal clonal analyses. For this, we condiQonally expressed her9DN and her9FL only in the boundary cells using the Gal4:UAS system, either with a UAS-bicistronic vector or the UAS-zebrabow2.0 system.

Nevertheless, the three referees raised concerns about the use of morphants. We fully agree that morpholinos may generate off-targets. However, I would like to stress as well that in zebrafish gene mutaQons have been shown to induce geneQc compensaQon (Rossi et al., 2015). Therefore, there is not a *bonafide* funcQonal experiment in this model system and thus, the need of several complementary funcQonal strategies. Since concerns about the morphant strategy were commonly raised by Referees, in this new version of the manuscript we provide an alternaQve loss-of-funcQon method. We have disrupted *her9* by using a CRISPR/Cas9-based approach that redundantly targets single genes and generates nearly complete gene disrupQon, consistently producing null phenotypes in F0 in zebrafish (Hoshijima et al., 2019; Wu et al., 2018). We recently set up this strategy in the lab, which provides the advantage that *her9* can be disrupted in the desired transgenic background in the F0. Using this strategy, we have been able to consistently confirm the main results of the study. As the Reviewers may know, this strategy is regularly known in the zebrafish field as CRISPANTS but since there is no common nomenclature yet, we called them *her9* sgRNAs injected embryos

© 2025. Published by The Company of Biologists under the terms of the Creative Commons Attribution License (https://creativecommons.org/licenses/by/4.0/).

(*her9* sgRNAs; see below for further experimental details).

The point-by-point responses to the Referees are as follows:

Reviewer 1

1. Reviewer 1 raises concerns about her9-MO experiments asking to demonstrate the specificity of the her9-MO by showing that her9-MO and her9DN produce similar phenotypes. This is not possible since both techniques are not comparable for the analysis of global effects in the boundary cell populaQon. her9-MO is injected at 1 cell stage and thus, all embryonic cells receive the her9-MO and the global phenotype can be assessed, whereas the her9DN is a DNA bi-cistronic construct under the control of UAS that is injected in Tg[BCP:Gal4] embryos. Thus, it will be condiQonally transacQvated only in boundary cells when Gal4 becomes acQve (around 24hpf, see (Hevia et al., 2022) and requires a clonal analysis. This is especially important when comparing boundary cell numbers in her9DN/her9FL experiments, since due to the sparse labelling, differences in numbers would be mostly caused by variability in injecQon/integraQon of the construct in each embryo. We believe that each funcQonal strategy is useful to address specific aspects of the same process, namely gene expression changes in the her9DN/her9FL clonal analysis and changes in the cell fate of the whole boundary populaQon in the her9-MO. Moreover, both strategies complement each other, since we have shown a clear increase in neuronal differenQaQon and decrease in the number of boundary cells per clone in the her9DN-zebrabow experiments (Figure 4) consistent with our results with the her9-MO.
   As an alternaQve and complementary LOF strategy we have generated *her9* sgRNAs injected embryos by redundantly targeQng *her9* by CRISPR/Cas9 to produce penetrant gene disrupQon in zebrafish in F0 (Figure S2E-I). We did it in the Tg[BCP:H2AmCherry;HuC:GFP] context (Figure 2G-I, G'-H', g'-h') to reproduce the neurogenic effects, and in the Tg[tp1:d2GFP] background (Figure S3D-F, D'-E', d'-e') to demonstrate that Notch-acQvity is repressed by Her9. We also showed a decrease of boundary Sox2 cells in her9 sgRNA embryos at 28hpf (Figure 2J-L, J-K', j'-k'). New results confirm the neurogenic effects and the phenotype on Notch-acQvity previously observed with the her9-MO, and are included in Figure 2 and Figure S3, respecQvely. InformaQon about *her9* CRISPANTS' design is included in Figure S2E-I.
2. The Reviewer asks about the p-value significance in Figure 2 and wonders about the sample size effect. We already provided the p-value informaQon in Figure 2 legend, showing that her9-MO embryos display an increase in boundary-derived neurons at 30hpf (control-MO 5.3% ± 4 n=6 vs. her9-MO 13.5% ± 5.7 n=9 embryos, p =0.007 [**], Welch's test) but not at 36hpf (control-MO 15.5% ± 8.2 n=6 vs. her9-MO 23.1% ± 5.5 n=9 embryos, p=0.08 [ns], Welch's test). The Reviewer is right that since there is some variability of *her9* morphants we might decrease p=0.08 by increasing the number of embryos. However, we have similar number of samples in other experiments with the her9-MO and we have been able to prove significance. Moreover, we have shown that *her9* is sustained in boundary cells from 18 to 30hpf and it decreases from 32hpf onward. Thus, we propose that this increase observed in boundary derived may be a consequence of the earlier neuronal differenQaQon caused by the morpholino. As in all experiments, we show an embryo with a phenotype representaQve of the mean, not of the extremes.
3. The Reviewer is right saying that expression of deltaD in Figure S3 appears more widespread in controls. We have reviewed the experiment and the differences in *deltaD* expression displayed in the original Figure are due to the fact that control and her9DN images correspond to different boundaries (as previously indicated in the figure legend). We selected these boundaries to show the most representaQve phenotype of the mean. However, we understand the issue raised by the reviewer and accordingly, we now display a control that corresponds to the same hindbrain boundary as the her9DN (Figure S3G-H, G'-H', g'-h').
4. He/she asks for clarification in Figures 3 and 4. We have rewriqen Figures 3 and 4 for beqer clarificaQons and added the informaQon about t0 and **r** within the text for a beqer comprehension. About the unchanged number of cells per clone between 36 and 48hpf in the her9FL condiQon (Figure 4F), we think there are two possibiliQes: either boundary cells can undergo a limited amount of PP divisions, or the phenotype observed at 48hpf is the

© 2025. Published by The Company of Biologists under the terms of the Creative Commons Attribution License (https://creativecommons.org/licenses/by/4.0/).

consequence of a temporary sustained her9FL expression as the Gal4 of the Tg[BCP:Gal4] only unQl 36hpf. We now comment about this issue and modify the main conclusion of this result in the manuscript.

5. He/she asks for clarification about EdU-incorporation experiments. As the Reviewer anticipated, we did a short EdU-pulse (1 hour) to assess the cell proliferative capacity. We have clarified it in the main text and in the Materials and Methods section.
6. It seems to be some misunderstandings about how we think that Her9 represses *cdkn1ca* at boundaries. In the manuscript, we propose that sustained Her9 constantly represses *cdkn1ca* expression in boundary cells. This repression would not allow Cdkn1ca to inhibit the acQvity of CyclinD1 protein (by blocking CyclinD1-Cdk complexes), and thus would permit a higher acQvity of CyclinD1 in boundary cells. We have beqer explained it to avoid misunderstandings. Dorsal views of embryos stained with *cdkn1a* upon injecQon of her9-MO are now provided in Figure 5O-P.
7. The Reviewer proposes to analyze the expression of *her9* in the *ccnd1*$^{pfu1/pfu1}$ mutants to demonstrate that *ccnd1* controls events in the boundaries. We claim that *ccnd1* is downstream of Her9 (not the opposite), since Her9 constantly represses *cdkn1ca* in boundary cells prevenQng it to inhibit the acQvity of CyclinD1. In this line, we have performed new experiments by injecQng her9-MO into the *ccnd1*$^{+/+}$ and *ccnd1*$^{pfu1/pfu1}$ embryos and analyzed the effects in boundary cell number. Upon downregulaQon of *her9* no differences in the decrease of boundary cells both in *ccnd1*$^{+/+}$ and *ccdn1*$^{pfu1/pfu1}$ embryos were observed (Figure 6H-L), indicaQng that no addiQve effects occur when both genes are disrupted. These results support the hypothesis that Her9 is upstream of CyclinD1 in the control of boundary cell proliferaQon.

8. We removed the Yap/Taz experiments as suggested by the Reviewer.

Reviewer 2

1. Reviewer 2 raises concerns about her9-MO experiments and propose to use the her9 mutant line sa43978. See general comments at the beginning of the leqer (second and third paragraphs). Thus, as an alternaQve and complementary LOF strategy we have generated *her9* sgRNAs injected embryos by redundantly targeQng *her9* by CRISPR/Cas9 to produce penetrant gene disrupQon in zebrafish in F0 (Figure S2E-I). We did it in the Tg[BCP:H2AmCherry;HuC:GFP] context (Figure 2G-I, G'-H', g'-h') to reproduce the neurogenic effects, and in the Tg[tp1:d2GFP] background (Figure S3D-F, D'-E', d'-e') to demonstrate that Notch-acQvity is repressed by Her9. We also showed a decrease of boundary Sox2 posiQve cells in *her9* sgRNA embryos earlier (28hpf; Figure 2J-L, J-K', j'- k'). New results confirm the neurogenic effects and the phenotype on Notch-acQvity previously observed with the her9-MO, and are included in Figure 2 and Figure S3, respecQvely. InformaQon about *her9* CRISPANTS' design is included in Figure S2E-I.
2. Reviewer raises concerns about the Tg[BCP:Gal4] line. The Tg[BCP:Gal4] line has been extensively characterized in Hevia et al, 2022 (see Suppl Figure 2 with legend from Hevia et al, 2022 at the end of this leqer). Briefly, in this transgenic line Gal4 was inserted in a well characterized cis-regulatory element of *sgca* (BCP) that drives gene expression to the boundary cell populaQon (Letelier et al., 2018). The specific hindbrain boundary genes and this transgenic line label the same cells. They do not cover the enQre transverse secQon of the hindbrain boundary, because there is the rhombic lip in the most dorsolateral domains, and neurons that at early stages do not derive from the boundaries are allocated in the ventral domain (Hevia et al, 2022). Therefore, *her9* is expressed in what is defined as the boundary cell populaQon labelled by boundary markers and the Tg[BCP:Gal4] line. To beqer demonstrate it to the reviewer, in the current version of the manuscript we provide staining of *her9* with a boundary marker such as *rfng* in the Tg[BCP:H2B-GFP] line in Figure S1. For beqer 3D comprehension we include dorsal and transverse views in Figure S1B-B'', with a magnificaQon of the framed corresponding boundary in dorsal view.
3. He/she wants us to beqer explain the method used for quanQficaQon of co-expression (Figure 2). We think that this is well explained in the Materials and Methods, but we have tried to improve our explanaQon including a drawing in Figure S2L-M, similar to the one displayed below. ROI contains all the stacks (black boxes) of each hindbrain whole

© 2025. Published by The Company of Biologists under the terms of the Creative Commons Attribution License (https://creativecommons.org/licenses/by/4.0/).

boundary (green) as shown in this dorsal view. The quanQficaQon is performed in all the XY planes contained within the ROI. The rectangles drawn in Figure 2 and Figure S3 are just to frame the displayed magnificaQons as examples.

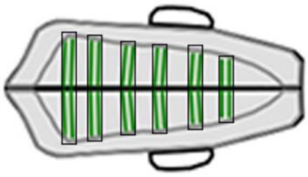

4. We think that the progenitor domain concept is well explained in the Materials and Methods. However, we provide a longer explanation together with examples of where cells are within the dorsoventral domain of the hindbrain (Figure S2L–M). We also included new immunostaining of neural progenitors (Sox2) and differentiated neurons (HuC) in the embryos injected with the UAS:zebrabow1.0 to show how the domains were defined at the initial and final time points (Figure S4A). Moreover, dotted lines as reference of the ventricular and mantle zone in the images of single clones were added (Figures 3 and 4). Finally, we have emphasized in the text how the presence of the apical contact was also a main criteria to assess cell fate in the multicolor clonal analysis.
9. We improved the staining of *her9* with *ccnd1* to beqer demonstrate their colocalizaQon at 26hpf but not at 36hpf (Figure 6C–D; Figure S5A). As we propose that Her9 is upstream of CyclinD1, we have performed new experiments by injecQng her9-MO into the $ccnd1^{+/+}$ and $ccnd1^{pfu1/pfu1}$ embryos and analyzed the effects in boundary cell number. Upon downregulaQon of *her9* no differences in the decrease of boundary cells both in $ccnd1^{+/+}$ and $ccdn1^{pfu1/pfu1}$ embryos were observed (Figure 6H–L), indicaQng that no addiQve effects occur when both genes are disrupted. These results support the hypothesis that Her9 is upstream of CyclinD1 in the control of boundary cell proliferaQon.
10. As indicated, the ZFIN genomic feature name of each published transgenic line was included in the Material and Method secQon.

Reviewer 3

1. It seems that there is a confusion about the different embryonic stages. We tried to convey this complexity by improving the wriQng in the revised version. Early stages such as 28-30hpf are those in which *her9* expression is enriched in the boundaries when they are not neurogenic, whereas rhombomeres are fully engaged in neurogenesis. Late stages such as 36hpf correspond to those in which boundaries are already engaged in neurogenesis and do not display sustained *her9* staining anymore. Unfortunately, we cannot analyze earlier stages with the Tg[BCP:Gal4] line because the acQvaQon of the transgene starts approximately at 24hpf and the paqern is not complete unQl 26-28hpf. In this line, the main reasons to look at late events are to clearly disQnguish between endogenous vs. ectopic *her9* expression driven by the UAS constructs, and to provide Qme for neurogenesis to proceed and properly assess the resulQng phenotype upon *her9* disrupQon. However, to answer the Reviewer's quesQon we have generated *her9* CRISPANTS in the Tg[BCP:H2AmCherry] line and analyzed changes in progenitor cells (Sox2) at 28hpf. These results back up our previous observaQons at 30hpf and are now included in Figure 2.
2. Reviewer raises concerns about the Tg[BCP:Gal4] line. The Tg[BCP:Gal4] line has been extensively characterized in Hevia et al, 2022 (see Suppl Figure 2 with legend from Hevia et al, 2022 at the end of this leqer). Briefly, in this transgenic line Gal4 was inserted in a well characterized cis-regulatory element of *sgca* (BCP) that drives gene expression to the boundary cell populaQon (Letelier et al., 2018). The specific hindbrain boundary genes and this transgenic line label the same cells. They do not cover the enQre transverse secQon of the hindbrain boundary, because there is the rhombic lip in the most dorsolateral domains, and neurons that at early stages do not derive from the boundaries are allocated in the ventral domain (Hevia et al, 2022). Therefore, *her9* is expressed in what is defined as the boundary cell populaQon labelled by boundary markers and the Tg[BCP:Gal4] line. To beqer demonstrate it to the reviewer, in the

© 2025. Published by The Company of Biologists under the terms of the Creative Commons Attribution License (https://creativecommons.org/licenses/by/4.0/).

current version of the manuscript we provide staining of *her9* with a boundary marker such as *rfng* in the Tg[BCP:H2B-GFP] line in Figure S1. For beqer 3D comprehension we include dorsal and transverse views in Figure S1B-B'', with a magnificaQon of the framed corresponding boundary in dorsal view. We have also provided beqer stainings of *ccnd1* and *her9*.

3. He/she asks for whole mount views for downregulaQon and GOF experiments in Figure 2. We believe transverse views provide a beqer boundary resoluQon and give us the posiQonal informaQon along the dorsoventral (DV) axis, which allow us to clearly show the fate (progenitor vs. neuron) of boundary cells. Also, due to the great number of panels and data already displayed in these figures, we think dorsal views would not help to follow the results. Moreover, the funcQonal condiQonal assays rely on the specific expression of her9DN and her9FL in the boundary cells by using the Gal4:UAS system with a bicistronic vector that will be condiQonally transacQvated only in boundary cells of Tg[BCP:Gal4] embryos when Gal4 becomes acQve (around 24hpf, for details of the transgenic line see Suppl Fig 2 (Hevia et al., 2022) included at the end of this leqer). Therefore, this a clonal experiment and the phenotype needs to be assessed in the boundary cells that express the constructs, not in the overall Qssue. Although we do not consider necessary to include the dorsal views because they do not bring any more informaQon, if she/he desires to see them, we can provide them to the Referee. In addiQon, we have beqer explained the assessment criteria used to analyze the phenotypes in Figure S2L-M.
4. The Referee is surprised with the slight increases in the percentage of boundary derived neurons upon her9-MO and effects in the her9DN. As addressed in the Discussion secQon, the moderate neuronal differenQaQon in the morphant could be explained by an early onset of asymmetric divisions. This is backed up by the data on Figure S3 in which we observed an early onset of Notch acQvity upon *her9* downregulaQon. We propose that high Her9 would keep Notch acQvity repressed in the boundaries, and upon Her9 downregulaQon Notch-acQvity would be on and promote asymmetric cell divisions. Thus, not all boundary cells differenQate, and some are kept as Notch acQve progenitors. Regarding the Reviewer's concern of the small porQon of differenQated her9DN cells: in the case of the her9DN-zebrabow data (Figure 4), there is a great shiV towards neuronal differenQaQon consistent with the higher percentage of progenitors in the her9FL. However, in the case of the single color her9DN analysis (Figure 2), we analyze the expression of a proneural (*neurog1*) and a neuronal differenQaQon gene (*neuroD4*) that are expressed at different Qmes during the neurogenesis process. Hence, the porQon of boundary cells detected in each experiment correspond to the cells in that specific step of neurogenesis. On the contrary, *sox2* expression domain covers all cells in the progenitor state.
5. We provide a more detailed explanation together with examples of where cells are within the hindbrain DV domain (Figure S2L-M). We also included new immunostaining of neural progenitors (Sox2) and differentiated neurons (HuC) in the embryos injected with the UAS:zebrabow1.0 to show how the domains were defined at the initial and final time points (Figure S4A). Moreover, dotted lines were added as reference of the ventricular and mantle zone in the images of single clones to provide context (Figures 3 and 4). Finally, Her9 manipulations lead to expected changes in cell morphology as the loss of apical contact in cells differentiated into neurons or the presence of the apical contact and the radial projection in progenitor cells. We have now emphasized this in the text.
6. The Referee asks for analysis of neurogenesis upon disrupQon of her9 at earlier stages.

Same answer as Point 1.

7. He/she is concerned about the number of boundary cells in the her9-MO embryos. The reducQon in the number of boundary cells is around 25% and we believe that the displayed embryo shows this reducQon. We selected an embryo falling in the average rather than an embryo with the strongest phenotype. We do not think that Her9 directly promotes *ccnd1* transcripQon as Her9 is a transcripQonal repressor, and this is the reason why we did not check *ccnd1* expression upon her9-MO injecQon. As we discussed in the manuscript, our hypothesis is that Her9 regulates *cdkn1a* in the boundaries, indirectly controlling CyclinD1 protein acQvity. We think that it is interesQng that the effect is not the same as in the hes1KO mice and we already discussed why we think it may be different in the Discussion secQon.
8. We understand the reviewer comment about providing EdU results without expression of *her9* or *fabp7a*. To strengthen this point, we have included the EdU-incorporaQon experiment at

© 2025. Published by The Company of Biologists under the terms of the Creative Commons Attribution License (https://creativecommons.org/licenses/by/4.0/).

48hpf to complement the previous one at 72hpf, with the staining of both *her9* and *fabp7a,* and we have quanQfied proliferaQng cells in different territories at 72hpf (Figure 7). Moreover, we showed that upon *her9* downregulaQon there was the loss of *sox2* expression at the transcripQonal level and now we have included the reducQon of the Sox2 protein domain (Figure 7G-J). It is interesQng the Referee's view that *her9* may transiQon from maintaining stemness in boundaries to promote radial glia fates in these lateral domains later. We propose that Her9 maintains the stemness of progenitors in both neuroepithelial progenitors (boundary cells) or radial glial cells at later stages (lateral domain). However, from our results, we could also propose that Her9 plays a role in promoQng radial glial fate at later stages.

9. Minor points: i) we do not know to what needed quanQficaQons the Reviewer refers to; ii) there is no clear manner to assess the efficiency of the *her9* constructs, but we always perform the staining of *her9* in the experiments with the bicistronic UAS constructs to analyze in the experimental condiQon only the H2BCitrine cells that express the *her9DN* or *her9FL*. However, we have confirmed the ectopic *her9DN*/*her9FL* expression by performing a double staining with probes that allow to disQnguish the ectopic *her9* (her9DN or her9FL) from the endogenous *her9* (Figure S2K-L); iii) all experiments with her9-MO were performed co-injecQng p53-MO as explained in Materials and Methods as shown to be enough to avoid off-target effects (Gerety and Wilkinson, 2011). However, we have demonstrated with the *her9* CRISPANTS that the phenotypes observed with the morphants are not caused by off target effects as both phenotypes were similar.

REFERENCES

Coomer, C. E., Wilson, S. G., Titialii-Torres, K. F., Bills, J. D., Krueger, L. A., Petersen, R. A., Turnbaugh, E. M., Janesch, E. L. and Morris, A. C. (2020). Her9/Hes4 is required for retinal photoreceptor development, maintenance, and survival. *Scientific Reports* 10, 11316–20.

Gerety, S. S. and Wilkinson, D. G. (2011). Morpholino artifacts provide pitfalls and reveal a novel role for pro-apoptotic genes in hindbrain boundary development. *Developmental Biology* 350, 279–289.

Hevia, C. F., Engel-Pizcueta, C., Udina, F. and Pujades, C. (2022). The neurogenic fate of the hindbrain boundaries relies on Notch3-dependent asymmetric cell divisions. *Cell Reports* 39, 110915.

Hoshijima, K., Jurynec, M. J., Shaw, D. K., Jacobi, A. M., Behlke, M. A. and Grunwald, D. J. (2019). Highly Efficient CRISPR-Cas9-Based Methods for Generating Deletion Mutations and F0 Embryos that Lack Gene Function in Zebrafish. *Developmental Cell* 51, 645-657.e4.

Letelier, J., Terriente, J., Belzunce, I., Voltes, A., Undurraga, C. A., Polvillo, R., Devos, L., Tena, J. J., Maeso, I., Rétaux, S., et al. (2018). Evolutionary emergence of the rac3b/rfng/sgca regulatory cluster refined mechanisms for hindbrain boundaries formation. *Proceedings of the National Academy of Sciences of the United States of America* 115,.

Rossi, A., Kontarakis, Z., Gerri, C., Nolte, H., Hölper, S., Krüger, M. and Stainier, D. Y. R. (2015). Genetic compensation induced by deleterious mutations but not gene knockdowns. *Nature* 524, 230–233.

Symonds, A. C., Buckley, C. E., Williams, C. A. and Clarke, J. D. W. (2020). Coordinated assembly and release of adhesions builds apical junctional belts during de novo polarisation of an epithelial tube. *Development* 147,.

Voltes, A., Hevia, C. F., Engel-Pizcueta, C., Dingare, C., Calzolari, S., Terriente, J., Norden, C., Lecaudey, V. and Pujades, C. (2019). Yap/Taz-TEAD activity links mechanical cues to progenitor cell behavior during zebrafish hindbrain segmentation. *Development* 146,.

Wu, R. S., Lam, I. I., Clay, H., Duong, D. N., Deo, R. C. and Coughlin, S. R. (2018). A Rapid Method for Directed Gene Knockout for Screening in G0 Zebrafish. *Developmental Cell* 46, 112-125.e4.

NOTE: Figure provided for reviewer has been removed. It showed Supplementary Figure 2 from **Hevia, CF, Engel-Pizcueta, C, Udina, F, Pujades C. The neurogenic fate of the hindbrain boundaries relies on Notch3-dependent asymmetric cell divisions. Cell Reports Jun 7; 39(10):**

© 2025. Published by The Company of Biologists under the terms of the Creative Commons Attribution License (https://creativecommons.org/licenses/by/4.0/).

110915. doi: 10.1016/j.celrep.2022.110915, 2022. We have removed unpublished data that had been provided for the referees in confidence.

Second decision letter

MS ID#: DEVELOP/2024/203164

MS TITLE: Her9 controls the stemness properties of hindbrain boundary cells

AUTHORS: Carolyn Engel-Pizcueta, Covadonga F Hevia, Adria Voltes, Jean Livet, and Cristina Pujades

I have now received all the referees reports on the above manuscript, and have reached a decision. The referees' comments are appended below, or you can access them online: please go to BenchPress and click on the 'Manuscripts with Decisions' queue in the Author Area.

The overall evaluation is positive and we would like to publish a revised manuscript in Development, provided that the referees' comments can be satisfactorily addressed. Please attend to all of the reviewers' comments in your revised manuscript and detail them in your point-by-point response. If you do not agree with any of their criticisms or suggestions explain clearly why this is so. If it would be helpful, you are welcome to contact us to discuss your revision in greater detail. Please send us a point-by-point response indicating your plans for addressing the referees' comments, and we will look over this and provide further guidance.

Reviewer 1

Advance summary and potential significance to field

As noted in my original review, this paper provides important new information about the role of the transcriptional repressor Her9 in the transition of rhombomere boundaries from sites of proliferation of progenitors to regions of neurogenesis and differentiation. Methods are incisive and include loss-of-function and misexpression assays, lineage-tracing, and analysis of patterns of proliferation and differentiation. This offers a useful complement to earlier studies of rhombomere centers.

Comments for the author

The authors have generally been responsive to reviewers' concerns and made a number of substantive changes to improve the manuscript. Most of my previous concerns have been satisfied. I must reiterate however, that conclusions regarding the relationships between her9, ckdn1ca, and ccnd1 need to be substantially moderated. Specific statements on page 11 and 13 "Her9 could control CyclinD1 activity in the boundaries through repression of cdkn1ca" and "Her9 promotes cell proliferation by repressing cdkn1ca", are not well supported and ignore potentially inconsistencies. First, it is likely that numerous steps separate her9 from regulation of cdkn1ca. Although data are consistent with a mechanism in which her9 represses neurog1, there are likely several additional steps leading to activation of cdkn1ca (e.g. neurog1 activates neurod1, which then activates expression of other transcription factors that cause differentiation of post-mitotic neurons). This progression takes time and involves too many steps to support the conclusion that Her9 transcriptionally represses ckdkn1ca. Second, the model that Her9 regulates CyclinD1 by repressing cdkn1ca (in the text and in the summary in Fig. 8) is muddled because it mixes regulatory relationships that occur on different levels (transcriptional vs. post-translational) and at different development stages. At early stages CyclinD1 is presumably regulated through normal cell division cycle regulators, and non-expression of cdkn1ca cannot be attributed to her9 without additional experimental support. Third, the statement that ccdn1 is an effector of her9 is likely too simple and neglects potential inconsistencies. Although there is certainly some overlap in expression of her9 and ccdn1, ccnd1 appears to have a much broader distribution, so many cells that express

© 2025. Published by The Company of Biologists under the terms of the Creative Commons Attribution License (https://creativecommons.org/licenses/by/4.0/).

ccnd1 do not appear to express her9. Hence the relationship between her9 and ccnd1 is likely indirect. There could be a non-autonomous role of her9 in regulating ccnd1, or her9 and ccnd1 may be co-regulated by a common upstream paracrine factor (e.g. Wnt). Such indirect/non-autonomous mechanisms are not considered at all, and repression of cdkn1ca seems misplaced since there are no data to support a direct role for her9.
Overall, regulatory relationships are not clear and should be discussed with greater caution and clarity.

Reviewer 2

Advance summary and potential significance to field

See the preamble to my original review - the advance and potential impact remains the same.

Comments for the author

The authors have adequately addressed my criticisms. I now recommend publication in Development without further revision.

Reviewer 3

Advance summary and potential significance to field

NA

Comments for the author

I am generally satisfied with the revision. Yet, inclusion of the new sgRNA line in other functional experiments, rather than only at the bottom of fig.2, would strengthen the results and the final conclusion.
As a general note, it is difficult to read the responses to the points raised by the reviewer without the full comments for context.

Second revision

Author response to reviewers' comments

Reviewer 1

1. *I must reiterate, however, that conclusions regarding the relationships between her9, ckdn1ca, and ccnd1 need to be substantially moderated. Specific statements on page 11 and 13 "Her9 could control CyclinD1 activity in the boundaries through repression of cdkn1ca" and "Her9 promotes cell proliferation by repressing cdkn1ca", are not well supported and ignore potentially inconsistencies. First, it is likely that numerous steps separate her9 from regulation of cdkn1ca. Although data are consistent with a mechanism in which her9 represses neurog1, there are likely several additional steps leading to activation of cdkn1ca (e.g. neurog1 activates neurod1, which then activates expression of other transcription factors that cause differentiation of post-mitotic neurons). This progression takes time and involves too many steps to support the conclusion that Her9 transcriptionally represses ckdkn1ca.*
We have clarified some of the statements regarding the proposed model of the control of boundary cell proliferation by Her9 and lowered down the tone as suggested. We agree with the reviewer that the increase in the expression of *cdkn1ca* in the *her9* morphants could be an indirect effect caused by the regulation of proneural/neuronal differentiation genes. However, as stated in the in the article, Hes transcription factors have been described to directly regulate the expression of cell cycle genes. Thus, it is also reasonable to propose that Her9 could be directly

© 2025. Published by The Company of Biologists under the terms of the Creative Commons Attribution License (https://creativecommons.org/licenses/by/4.0/).

repressing *cdkn1ca* expression and I think that it is fair to argue this in the Discussion section.

2. The model that Her9 regulates CyclinD1 by repressing cdkn1ca (in the text and in the summary in Fig. 8) is muddled because it mixes regulatory relationships that occur on different levels (transcriptional vs. post-translational) and at different development stages. At early stages CyclinD1 is presumably regulated through normal cell division cycle regulators, and non-expression of cdkn1ca cannot be attributed to her9 without additional experimental support. Third, the statement that ccdn1 is an effector of her9 is likely too simple and neglects potential inconsistencies.
We agree that regulation of the cell cycle is complex, and many other players participate in its regulation. We do not claim this as the solely mechanism; rather, we propose a putative regulatory model. Indeed, we have stated in the Discussion how future studies should be performed to continue dissecting the control of cell cycle in hindbrain boundary cells.

3. Although there is certainly some overlap in expression of her9 and ccdn1, ccnd1 appears to have a much broader distribution, so many cells that express ccnd1 do not appear to express her9.
ccnd1 expression is indeed broader in the whole hindbrain, but we consistently showed that it largely overlaps with *her9* in boundary cells.

4. Hence the relationship between her9 and ccnd1 is likely indirect. There could be a non-autonomous role of her9 in regulating ccnd1, or her9 and ccnd1 may be co-regulated by a common upstream paracrine factor (e.g. Wnt). Such indirect/non-autonomous mechanisms are not considered at all, and repression of cdkn1ca seems misplaced since there are no data to support a direct role for her9. Overall, regulatory relationships are not clear and should be discussed with greater caution and clarity.
We are proposing that the regulation of Cyclin D1 by Her9 is through the repression of cell cycle arrest gene, therefore indirect. Although we explored *cdkn1ca*, we cannot rule out that other cell cycle regulators might be involved. Our data shows that when we combine *her9* and *ccnd1* loss-of-function we obtain the same decrease in the boundary cell numbers as the single loss of each gene. This suggest that Her9 promotes hindbrain boundary cell cycle progression potentially through the regulation of CyclinD1. We cannot discard other mechanisms cooperating in the control of proliferation as mentioned by the reviewer. For this reason, we discuss in the article how other mechanisms as Yap/Taz-Tead activity could be synergically controlling this process.

Reviewer 3

1. I am generally satisfied with the revision. Yet, inclusion of the new sgRNA line in other functional experiments, rather than only at the bottom of fig.2, would strengthen the results and the final conclusion.
As proposed by the Reviewer, we added another Her9 loss-of-function approach and analyzed some of the main observed phenotypes with the CRISPR-Cas9 based *her9* sgRNA technique. We have demonstrated that *her9* sgRNA injected embryos display a similar effect in neuronal differentiation (Figure 2) and in Notch-activity (Figure S3) than with the other techniques (her9MO and her9DN). Moreover, we have shown that *her9* sgRNA injections result in the effects in the opening of the hindbrain ventricle as her9MO in Figure S2. Therefore, these results reinforce the ones observed upon her9MO injections and the different conditional expression of *her9DN* constructs (using a UAS-bicistronic vector or the zebrabow2.0 system) as reliable approaches to study Her9's function. We believe that each of the four functional approaches allow us to address distinct aspects of the Her9's function, and that all results are complementary and support our claims about Her9 function.

2. As a general note, it is difficult to read the responses to the points raised by the reviewer without the full comments for context.
We apologize with the Reviewer and thank him/her for being so patient. We just wanted to avoid being very repetitive.

<u>Third decision letter</u>

© 2025. Published by The Company of Biologists under the terms of the Creative Commons Attribution License (https://creativecommons.org/licenses/by/4.0/).

MS ID#: DEVELOP/2024/203164

MS TITLE: Her9 controls the stemness properties of hindbrain boundary cells

AUTHORS: Carolyn Engel-Pizcueta, Covadonga F Hevia, Adria Voltes, Jean Livet, and Cristina Pujades

I am delighted to tell you that your manuscript has been accepted for publication in Development, pending our standard publication integrity checks.

© 2025. Published by The Company of Biologists under the terms of the Creative Commons Attribution License (https://creativecommons.org/licenses/by/4.0/).
